# A mixed-methods nutrition, water, sanitation and hygiene knowledge, attitudes, and practices survey of IDPs, returnees, and host community members in four counties of Jonglei state, South Sudan

**DOI:** 10.1186/s41043-025-00789-3

**Published:** 2025-02-25

**Authors:** Lynn Lieberman Lawry, Rachel Gabor, Jacques Katele, Lazarus Baak Madut, Katina Sommers, David Manuel, Claire Nadolski, Mounir Lado, Tracey Perez Koehlmoos, William Clemmer

**Affiliations:** 1 Preventive Medicine and Biostatistics Department, Uniformed Services University, 4301 Jones Bridge Rd., Bethesda, MD 20814-4799 USA; 2 University of Juba, University Street., Juba Town, South Sudan; 3 IMA World Health, 1730 M St NW #1100, Washington, DC 20036 USA; 4 IMA World Health South Sudan, Heran Office Premises, Hai Cinema, Plot #82, Juba Stadium Road, 3Rd Floor, Juba, South Sudan; 5 IMA World Health DRC, 1 Av Tissakin, Kinshasa NGALIEMA, Kinshasa, Democratic Republic of the Congo

**Keywords:** Nutrition, Water sanitation and hygiene, WASH, South Sudan, Conflict health

## Abstract

**Background:**

The humanitarian community in South Sudan estimates that 9.4 million people need humanitarian assistance in 2023. Prior data is unlikely to reflect the current health situation of Jonglei state given the influx of internally displaced persons (IDPs) and returnees.

**Methods:**

We conducted a cross-sectional, randomly sampled, mixed-methods, population-based household study during a 4-week period in June-July 2023 in ten bomas in four counties of Jonglei, South Sudan. Snowball sampled qualitative interviews were used for triangulation of quantitative data. The study was conducted to understand nutrition and water, sanitation, and hygiene knowledge, attitudes, and practices of IDPs, returnees and the host communities.

**Results:**

A total of 859 households consented to the study (586 females and 273 males) with a response rate of 96% among females and 94% among males. Forty-two percent of households identified three or more strategies to prevent starvation or malnutrition. Up to a fifth of households were unable to identify any strategies to prevent starvation or malnutrition with the host community having the highest rate (20.2%) when compared to IDPs (13.4%; *p* = 0.018) or returnees (10.4%; *p* = 0.006). Only 34% of respondents could name three warning signs for when a child with diarrhea should be seen by a skilled provider. Only 26% of females exclusively breastfeed. Higher odds of breastfeeding included IDPs (aOR 1.11; 95% CI 1.01 to 1.03; p = 0.038), those educated on breastfeeding (aOR 1.18; 95% CI 1.03 to 1.35; *p* = 0.015) and those with positive nutrition attitudes (aOR 1.32; 95% CI 1.09 to 1.59; *p* = 005). Estimated household water usage ranged from 7.9 to 10.1 L/person/day. Based on qualitative triangulation, both nutrition and WASH traditional beliefs hinder adequate nutrition and create barriers to improvements in WASH.

**Conclusion:**

Evidence based nutrition interventions should be the priority and include, at a minimum, micronutrient and vitamin supplementation, food fortification and adequate supplemental food for reproductive age females. Immediate and exclusive breastfeeding messaging should be a priority. Improved flood resistant sources for clean water, clean covered water storage container distribution, improved sanitation, oral rehydration solution (ORS) and zinc distribution and education are necessary to decrease diarrheal rates.

**Supplementary Information:**

The online version contains supplementary material available at 10.1186/s41043-025-00789-3.

## Introduction

South Sudan continues to face deteriorating humanitarian conditions [1]. Ongoing violence, after failed peace agreements, created critical economic and social impacts including a drastic decrease in the national budget for health and increasing inflation [1]. The situation is worsened by intercommunal violence, conflict, access constraints, public health challenges (e.g. measles and cholera), flooding, and localized droughts [2]. There are an estimated 2.5 million internally displaced persons (IDPs) across the country—50% of whom are children, with more than one million South Sudanese refugees currently hosted in neighboring countries [3]. The humanitarian community in South Sudan estimates that 9.4 million people need humanitarian assistance in 2023, which is 76% of South Sudan’s population [1].

South Sudan ranks 106 out of 121 countries on the Global Hunger Index. [4] Stunting is prevalent among 31.3% of children under five years of age in South Sudan, which is higher than the average for the Africa region (30.7%) [4]. Nearly 23% of children under five years of age have wasting, which is much higher than the average for the Africa region (6.0%) and among the highest in the world [4]. Every year, there are 1.7 billion cases of diarrhea among children younger than five years putting them at increased risk for malnutrition [5]. Thus, the provision of safe water, sanitation, and hygiene (WASH) to affected populations is necessary for dignity, communicable disease control and improved nutritional status [5]. 

Nutrition and WASH are intricately linked [6, 7]. Inadequate WASH increases the risk of undernutrition, especially among children ≤ 2 years. [7] Nearly half of malnutrition cases are linked to recurrent diarrhea and or intestinal infections due to the lack of access to sufficient safe drinking water and poor sanitation facilities [6]. The lack of adequate WASH puts millions of people at risk for diseases, many of which are water- and hygiene-related and found where there are poor WASH practices [8]. Ayod is one of the counties in Jonglei most affected by the flooding even during the dry season, due to climate change impacts [9]. Without safe water points, often due to submerged boreholes, communities are forced to use stagnant water sources. (Supplemental Fig. 1) Prevention is key. Increasing access to safe drinking water and improved sanitation services can prevent many diarrheal deaths.

Nine percent of the 5.8 million deaths of children younger than five years are from diarrhea, mostly in low- and middle-income countries [5]. Only two percent of South Sudanese households have access to water and 68.7% of the household population has access to improved drinking water sources; 78% in Jonglei [10]. Improved sanitation facilities are defined as flush toilets connected to sewage systems, septic tanks or pit latrines, ventilated improved pit latrines and pit latrines with slabs, and composting toilets [11]. Only seven percent of household population use improved sanitary facilities, compared to 64 percent who practiced open defecation [10]. Safe disposal of children’s waste occurs in fewer than 16 percent [10]. 

Undernutrition continues to be a major health problem in South Sudan [12]. National-level data show that 15.1% of children aged younger than 5 years are stunted, with 3.9% severely stunted [13]. Nationally (2019), women’s nutrition status is also poor, with 38.2% of women of childbearing age (excluding pregnant women) being underweight (body mass index less than 18.5%) [13]. The under-five mortality rate (U5MR) in South Sudan remains high at 96.2 deaths/1000 live births with boys having a higher U5MR than girls (101 and 91 deaths/1,000 live births, respectively) [14]. 

Women’s nutritional status before, during, and after pregnancy affects their own well‐being, mortality risk and their children’s health outcomes [15]. There are global nutritional commitments to focus on the first 1,000 days of life for improving nutrition outcomes among children [15]. There are ten high impact nutrition-specific interventions, that if scaled up to 90% coverage across the lifecycle and through the first 1000 days can result in reducing stunting prevalence by 20% and wasting prevalence by 60% [16]. These ten interventions are implemented in South Sudan to some degree and include periconceptual, pregnancy and lactation folic acid supplementation, maternal balanced energy protein supplementation, maternal calcium supplementation, multiple micronutrient supplementation in pregnancy, promotion of breastfeeding, appropriate complementary feeding, vitamin A administration and preventive zinc supplementation in children 6–59 aged months, and screening for and management of severe acute malnutrition and moderate acute malnutrition [17]. 

Conflict and insecurity, loss of functional health centers, incomplete data in the district health information system 2.0 (DHIS2), and increased flooding and displacements necessitates an understanding of the current state of nutrition, water, sanitation, and hygiene using a knowledge, attitudes and practices (KAP) survey among IDPs, returnees and host communities in remote counties of Jonglei state hosting many of the IDPs and returnees [10, 18]. 

## Methods

### Study design

We conducted a cross-sectional, randomly sampled, mixed-methods, population-based household study during a 4-week period in June-July 2023 in ten bomas in Ayod, Nyirol, Fangak, and Pigi counties of Jonglei, South Sudan. (Table S1) The study was conducted to understand the knowledge, attitudes, and practices of IDPs, returnees and the host communities relative to nutrition and WASH quantitatively with qualitative interviews used for triangulation that occurred within each boma simultaneously We assumed 50% of females practiced exclusive breastfeeding. The sample size required to estimate nutrition and WASH KAPs through a random sample with 95% confidence (margin of error of ± 5%) was 770 households assuming a design effect of two. To account for refusals and those not at home, we increased the sample size to 900 households: 225 household surveys per payam and population-based to each boma, villages, and settlements within payams. Eligibility criteria included household females and males willing to consent to the study, were ≥ 18 years of age or emancipated minors with children less than 5 years of age. Males were sampled from every 5th household interviewed. Adult (≥ 18 years) females and males were selected via systematic random sampling among households and sampled according to a modified World Health Organization (WHO) Expanded Programme on Immunisation (EPI) Method [19]. Security constraints, distance, and flooding required using purposive and systematic transect selection [20]. Transects were selected either from the village center (or a village main accessible point) or from different points of main roads [20]. 

### Household sampling

#### Quantitative household interviews

Twelve local data collectors were trained and supervised by three experienced field researchers. Surveyors conducted one-on-one, anonymous interviews with a randomly selected male or female household members in a private setting. If no one met the criteria or if no one was home, the next household to the right was surveyed. Interviews took approximately 30–40 min and were conducted in Nuer, Dinka, and other local languages using a standardized survey form on KoboToolBox, an open-source platform for collecting, managing, and visualizing data in challenging environments [21]. 

#### Qualitative interviews

In-depth qualitative interviews were used to triangulate issues identified in the quantitative instrument to identify common themes that are involved in nutrition and WASH context in the four target counties of Jonglei state. Convenience, purposeful and snowball sampling (non-probability sampling) methods were used to allow flexibility in accessing individuals who might be important for informing the quantitative data. Trained data supervisors experienced in qualitative sampling completed the interviews as the quantitative survey was on-going in the selected boma. Interviews, conducted in Nuer, Dinka, and other local languages were typed into Kobotoolbox forms in English by data managers who were fluent in multiple languages. A total of 64 key informants (21 females; 43 males) were interviewed. Interviews included community members, leaders, beneficiaries, non-governmental organization (NGO) personnel, Ministry of Health (MOH), United Nations, World Bank, healthcare providers, traditional birth attendants (TBAs), community health workers (CHWs), and traditional healers such as spirit masters and witch doctors. Only interviews that triangulated nutrition and WASH quantitative themes were utilized.

### Data collector training

We developed a standardized protocol for training study supervisors and data collectors that is used in and adapted to a variety of settings [16]. Study supervisors were trained over 3-days to include data collector supervision, study methodology, qualitative sampling and interviewing. All data managers were hired based on previous qualitative training and experience. Trained, qualified, Nuer and Dinka speaking supervisors trained locally hired data collectors in a coordinated program of classroom teaching and experimental role-play followed by several days of field observation and testing [22]. Observed role-play of survey questions and field testing was designed to both ensure functional equivalence of the questions across the local languages as well as adjust for cultural issues. Our data collectors are literate and have at least a high school level of education. The style of the program is participatory and interactive with the trainers. At the end of the training, trainees are tested on content and their ability to conduct an interview using a mock survey that incorporates difficulties they may encounter in the field [22]. A good training curriculum enhances the future interviewer’s understanding of the survey and ability to translate it into any language they will be using.

### Instruments

#### Quantitative instrument

We used survey-research techniques modeled on epidemiological instruments previously developed, implemented, and validated for South Sudan [20]. Instruments (quantitative and qualitative) were developed in collaboration with IMA World Health and the South Sudan Ministry of Health to capture the nuance and social norms in regard to the survey objectives. The quantitative survey was developed for the Jonglei context to elucidate nutrition and WASH knowledge, attitudes, and practices among the survey population of IDPs, returnees, and the host community. The instrument contained 103 questions on nutrition and WASH questions. (Additional File 1).

#### Qualitative instrument

The qualitative instruments contained up to 18 open-ended questions relating to health, nutrition, WASH, cultural norms, traditional practices, and community needs. (Additional File 2 and 3) Two different interview guides were adapted from previously developed, implemented, and validated guides for an area similar to Jonglei [20]. 

### Analytical methods

#### Quantitative

Quantitative household data were analyzed using R version 4.1.1 with the survey and weights packages [23]. Analysis involved the estimation of weighted population means and percentages by displaced type (IDP, returnee, host community) with 95% confidence intervals. Comparisons by displacement type were made using a weighted t-test and weighted chi-squared test, as appropriate, and considering p-values as significant when less than 0.05. Weighting was necessary to ensure that surveyed households were representative of the relative size of each boma within the project area. Missing data was minimal for relevant variables (< 2%) and dropped from analyses. Adjusted odds ratios were estimated from a weighted logistic regression model with knowledge or attitudes as the outcome of interest and displaced type, age, sex, education, underage marriage, and a priori variables of interest as factors in the model.

#### Qualitative

Qualitative narratives were directly transcribed in English into KoboToolBox. Narratives from the key informant interviews were summarized and compiled into a master Excel spreadsheet by outcomes and questions on the instruments. Data were analyzed using conceptual content analysis, whereby researchers identify concepts or themes that are quantified and examined for triangulation of these data for selected themes in the quantitative data [24] Themes, developed collaboratively with local field staff to ensure culturally and contextually appropriate interpretation of the analysis, were hand-coded, analyzed, categorized by the first author, and reviewed by data managers for concurrence to limit bias.

## Results

### Demographics

A total of 859 households consented to the study (586 females and 273 males) with a response rate of 96% among females and 94% among males. (Additional File IV -Table S2) Female respondents were on average 31.0 (95% CI 29.9 to 32.1) years and males 36.0 (95% CI 34.6 to 37.4) years. Respondents were mostly of the Neur and Dinka ethnicity, married, had no formal education, illiterate, and reported their occupations to be farmers, herders, or fishermen. Only 7.5% of females and 17% of males were able to read the directions on an ORS packet in Neur, Dinka, or Juba Arabic. Fifteen percent of females and 22% of males reported a disability.

### Nutrition knowledge

Only 39% of respondents had training on breast or complimentary feeding. (Table 1) IDPs were less knowledgeable than host community members [30.4% (95% CI 24.8–36.6%) vs. 45.0% (95% CI 39.5–50.7%); *p* < 0.001] Less than 50% of households identified three or more strategies to prevent starvation or malnutrition. Up to a fifth of households were unable to identify any strategies to prevent starvation or malnutrition with the host community having the highest rate (20.2%) when compared to IDPs (13.4%; *p* = 0.018) or returnees (10.4%; *p* = 0.006).

**Table 1:** Nutrition and WASH knowledge among 859 households in Jonglei South Sudan, June-July 2023

	N	All	IDPs	Returnees	Host Community	p-Value^a^	*p*-Value^a^
		Weighted % (95% CI)	Weighted % (95% CI)	Weighted % (95% CI)	Weighted % (95% CI)	IDP vs Host Community	Returnee vs Host Community
Nutrition Knowledge							
Educated on breastfeeding or complimentary feeding^b^	729	39.4 (35.7–43.2)	30.4 (24.8–36.6)	42.4 (33.9–51.3)	45.0 (39.5–50.7)	< 0.001	0.603
Identified strategies to prevent starvation or malnutrition							
None	859	16.0 (13.6–18.8)	13.4 (9.8–18.1)	10.4 (6.3–16.8)	20.2 (16.3–24.8)	0.018	0.006
3 or more	859	41.6 (38.1–45.1)	43.4 (37.5–49.4)	36.4 (28.9–44.6)	42.4 (37.4–47.6)	0.800	0.193
Learned signs from	716						
Clinic		45.1 (41.3–49.0)	39.0 (33.0–45.4)	49.0 (40.5–57.6)	48.2 (42.5–54.0)	0.026	0.871
TBA		21.0 (18.0–24.4)	24.1 (18.9–30.1)	19.0 (13.1–26.9)	19.5 (15.3–24.6)	0.186	0.902
CHW		24.8 (21.7–28.2)	25.5 (20.3–31.5)	21.1 (14.9–28.9)	26.0 (21.3–31.3)	0.889	0.260
Relative		44.6 (40.8–48.5)	54.6 (48.1–60.9)	40.9 (32.7–49.6)	38.4 (32.9–44.2)	< 0.001	0.610
Mother/mother-in-law		27.1 (23.8–30.7)	26.5 (21.2–32.7)	29.8 (22.5–38.3)	26.3 (21.5–31.8)	0.954	0.442
Media^c^		2.4 (1.4–3.8)	1.2 (0.4–3.6)	2.5 (0.8–7.4)	3.3 (1.8–6.0)	0.094	0.638
WASH Knowledge							
Identified reasons for handwashing							
None	859	5.6 (4.2–7.5)	3.7 (2.0–6.9)	4.2 (1.8–9.5)	7.6 (5.2–10.9)	0.033	0.152
3 or more	859	61.5 (58.0–64.9)	69.5 (63.6–74.8)	61.8 (53.6–69.5)	55.4 (50.2–60.5)	< 0.001	0.171
Hand washing knowledge gained from:	810						
Clinic		41.8 (38.3–45.4)	37.1 (31.5–43.1)	47.3 (39.1–55.7)	43.1 (37.8–48.4)	0.121	0.378
TBA		16.9 (14.3–19.8)	21.9 (17.2–27.5)	14.1 (9.2–21.0)	14.2 (10.9–18.5)	0.010	0.967
CHW		24.5 (21.5–27.7)	25.0 (20.0–30.7)	21.0 (15.0–28.6)	25.6 (21.3–30.5)	0.853	0.270
Relative		43.2 (39.6–46.8)	51.2 (45.1–57.3)	39.2 (31.4–47.6)	38.7 (33.6–44.1)	0.001	0.919
Mother/mother-in-law		28.3 (25.1–31.7)	26.3 (21.2–32.1)	34.4 (27.0–42.8)	27.3 (22.7–32.3)	0.776	0.105
Media^c^		3.9 (2.7–5.6)	3.3 (1.6–6.4)	5.7 (2.8–11.1)	3.6 (2.1–6.3)	0.809	0.289
Able to name 3 warning signs a child with diarrhea should be seen	859	33.9 (30.6–37.3)	40.4 (34.6–46.4)	33.7 (26.5–41.9)	29.2 (24.8–34.1)	0.002	0.299
Learned these signs from:	694						
Clinic		41.4 (37.6–45.3)	37.5 (31.5–43.9)	50.1 (41.6–58.7)	40.5 (34.7–46.5)	0.473	0.059
TBA		21.6 (18.5–25.0)	23.4 (18.3–29.5)	19.2 (13.2–27.0)	21.1 (16.6–26.5)	0.514	0.643
CHW		25.7 (22.5–29.2)	24.6 (19.5–30.6)	20.2 (14.2–28.0)	29.3 (24.2–34.9)	0.215	0.045
Relative		49.8 (45.8–53.7)	59.9 (53.4–66.0)	42.2 (33.9–50.9)	44.8 (38.9–50.9)	< 0.001	0.608
Mother/mother-in-law		28.7 (25.2–32.4)	27.8 (22.3–34.0)	29.9 (22.6–38.3)	28.8 (23.7–34.6)	0.789	0.824
Media^c^		2.2 (1.3–3.7)	1.2 (0.4–3.6)	4.3 (2.0–9.1)	2.0 (0.8–4.8)	0.435	0.171

*CI* confidence interval

^a^Categorical variables: Pearson ^2^ test; Continuous variables: two tailed t-test for means

^b^Percentages are calculated out of respondents with children under 5 years old in their household

^c^Media includes phone, TV, written material (newspaper, fliers, billboards), and mobile messages

### WASH knowledge

Nearly 62% of all respondents could identify three reasons for handwashing. When compared to the host community, IDPs had statistically significant higher rates of identifying three reasons for handwashing (*p*<0.001). (Table 1) Youth (aOR 1.03; 95% CI 1.01 to 1.06; *p *= 0.007), and those in Magok-Panyang boma (aOR 1.03; 95% CI 1.00 to 1.07; *p* = 0.048), had 3% higher odds of identifying three or more reasons for handwashing. (Table 1; Additional File IV -Table S3) Respondents with <4 years of formal education (aOR 0.95; 95% CI 0.92 to 0.98; *p *= 0.02), or those with a pit latrine (aOR 0.95; 95% CI 0.92 to 0.98; *p *= 0.004) in the household had a 5% decrease, and those with handwashing stations in the household (aOR 0.89; 95% CI 0.84 to 0.95; *p *= 0.001), or living in the boma of Mat (aOR 0.89; 95% CI 0.82 to 0.97; *p *= 0.006), had an 11% decrease in the odds of identifying three or more reasons for handwashing. (Additional File IV -Table S3) Only 34% of respondents could name three warning signs for when a child with diarrhea should be seen by a skilled healthcare provider. IDPs had a higher proportion of households that could identify these warning signs when compared to the host community (*p *= 0.002) (Table 1).

### Nutrition attitudes

Agreement with various nutrition statements ranged from 91% to 99% among all groups. (Table 2) However, IDPs when compared to host community households agreed less that babies should be exclusively breast fed for the first six months (97% vs. 99% *p* = 0.02) and that infants and children should be weighed on a regular basis at the clinic (80% vs 97%; *p* < 0.001).

**Table 2:** Nutrition and WASH Attitudes among 859 Households in Jonglei South Sudan, June-July 2023

	N	All	IDPs	Returnees	Host Community	*p*-Value^a^	*p*-Value^a^
		Weighted % (95% CI)	Weighted % (95% CI)	Weighted % (95% CI)	Weighted % (95% CI)	IDP vs Host Community	Returnee vs Host Community
Nutrition Attitudes—Agreement							
It is good to feed a child different types of food each day	816	91.8 (89.6–93.7)	90.0 (85.5–93.2)	91.7 (85.5–95.3)	93.3 (90.0–95.5)	0.128	0.527
Early breastmilk protects newborn babies from infection	823	91.7 (89.5–93.6)	91.7 (87.5–94.6)	89.4 (82.9–93.7)	92.7 (89.3–95.1)	0.630	0.221
Babies should be breastfed exclusively for the first 6 months	828	98.6 (97.4–99.2)	97.1 (94.1–98.6)	99.3 (94.9–99.9)	99.4 (97.6–99.8)	0.020	0.872
Infants and children should be weighed on a regular basis at the clinic	793	91.0 (88.6–92.9)	80.4 (74.7–85.1)	95.3 (89.9–97.9)	96.6 (94.0–98.1)	< 0.001	0.480
WASH Attitudes—Agreement							
Consumption of safe and enough water can prevent waterborne diseases	831	94.5 (92.6–95.9)	93.6 (89.9–96.0)	95.6 (91.1–97.8)	94.8 (91.7–96.7)	0.506	0.703
Defecating near a water source can cause contamination	831	96.2 (94.7–97.4)	94.2 (90.6–96.4)	97.6 (94.1–99.0)	97.3 (94.9–98.5)	0.044	0.834
Animal dung, if not properly managed, causes health problems	759	88.2 (85.4–90.4)	87.9 (82.8–91.7)	89.5 (82.5–93.9)	87.9 (83.8–91.0)	0.977	0.620
Washing hand after using latrine prevents diarrheal diseases	826	97.5 (96.1–98.5)	97.0 (94.0–98.5)	98.5 (94.1–99.6)	97.6 (95.2–98.8)	0.660	0.508
Diarrheal diseases are caused by poor personal hygiene and sanitation	829	98.0 (96.7–98.8)	97.1 (94.0–98.6)	97.8 (93.4–99.3)	98.8 (96.8–99.5)	0.117	0.411

Decreased odds for positive nutrition attitudes included those with children treated for malnutrition (aOR 0.93; 95% CI 0.88 to 0.98; *p* = 0.008) or those living in Kurwai (aOR 0.63; 95% CI 0.55 to 0.72; *p* < 0.001) and Magok-Panyang (aOR 0.44; 95% CI 0.40 to 0.48; *p* < 0.001) bomas. (Table 3; Additional File IV-Table S4).

**Table 3:** Statistically significant Weighted Odd Ratios of WASH and Nutrition KAP Survey Results among 859 Households in Jonglei South Sudan, June–July 2023

	Weighted OR	95% CI	*p*-value
*Knowledge of Handwashing*			
Younger age (16–25 years)	1.03	1.01–1.06	0.007
Less than 4 years of formal education	0.95	0.92–0.98	0.002
Pit latrine in the household	0.95	0.92–0.98	0.004
Handwashing station in the household	0.89	0.84–0.95	0.001
Boma			
Magok-Panyang	1.03	1.00–1.07	0.048
Mat	0.89	0.82–0.97	0.006
*Positive Nutrition Attitudes*			
Children treated for malnutrition	0.93	0.88–0.98	0.008
Boma			
Kurwai	0.63	0.55–0.72	0.000
Magok-Panyang	0.44	0.40–0.48	0.000
Mat	1.08	1.03–1.14	0.003
*Exclusive Breastfeeding*			
IDP vs. Host	1.11	1.01–1.23	0.038
Educated on breastfeeding	1.18	1.03–1.35	0.015
Positive nutrition attitudes	1.32	1.09–1.59	0.005
Boma			
Korfulus	1.37	1.15–1.65	0.001
Magok-Panyang	1.46	1.12–1.92	0.006
Mat	1.41	1.13–1.77	0.003
Chortbora	0.72	0.56–0.94	0.017
Pigi	1.40	1.16–1.70	0.001
Tock	0.78	0.64–0.95	0.015

Adjusted odds ratios were estimated using a GLM with survey weights

### Traditional nutrition beliefs

*“There is a belief that having sex during the breastfeeding period will cause malnutrition of the newborn and the decision of when the breastfeeding period is over is not driven by health but by men who have decision authority and are unwilling to wait for 6 months of exclusive breastfeeding before sexual intercourse can resume.”* (DM1; Additional File IV -Table S5) Local nutritional beliefs, especially among the Dinka, are that mothers should eat chicken after delivery, to produce more milk. Beliefs also forbid eating ground nuts for up to 4 weeks after delivery. (DM3; Additional File IV-Table S5) It is believed that children fed eggs or chicken will not talk or will develop hypertension. (DM1,3; Additional File IV -Table S5).

### WASH attitudes

Except for the lack of agreement that animal dung, if not properly managed causes health problems, respondents across all groups had positive attitudes about WASH topics (94–98%). (Table 2) Across the survey areas, there is a common belief that *“infants should not be washed for seven days after birth because it will make them more susceptible to infection or malnutrition.”* (DM1; Additional File IV-Table S4) This prohibits adequate cord care and does not allow for appropriate cleaning after defecation or urination leading to infection risk. In addition, *“women do not wash for seven days after birth and are required to sit in the sand until they stop bleeding.”* (DM1; Additional File IV-Table S4).

### Nutrition practices

#### Breastfeeding practices

Overall, 95% of females stated they breastfed their last baby with no differences among groups. (Table 4) A maternal medical problem (23%) was the most common reason for not breastfeeding. IDPs, compared to the host community, had much higher rates of “breastfeeding difficulty” as a reason for not breastfeeding [51.6% (95% CI 16.3%-85.4%) vs. 7.3% (95% CI 1.0%-37.9%); *p* = 0.007]. Immediate breastfeeding rates (within one hour of delivery) were high across all groups (range 91%-97%). Only a quarter of females exclusively breastfed (≥ 6 months). Higher odds of breastfeeding included IDPs (aOR 1.11; 95% CI 1.01 to 1.03; *p* = 0.038), those educated on breastfeeding (aOR 1.18; 95% CI 1.03 to 1.35; *p* = 0.015) and those with positive nutrition attitudes (aOR 1.32; 95% CI 1.09 to 1.59; *p* = 005). (Table 3), Additional File IV-Table S6) Complimentary feeding started before the recommended six months with a variety of supplements given. (Table 4) Cow’s milk (85%), the most common supplement was more prevalent among the host community (93%) and much less prevalent among IDPs (73%; *p* < 0.001). (Table 4) Water, sorghum/rice porridge, or goat’s milk were also given as supplements. Powdered milk supplements, but not infant formula, were readily available in the markets in the survey areas. (DM1-4, Additional File IV -Table S4) Consequently, 17% of all respondents stated they are using these supplements with 31% of returnees using powdered milk supplements for infant feeding. A fifth of female (21.7%) stated there was no one to help with breastfeeding difficulties. And although 78% of women stated there was breastfeeding help, the help included mother or mother-in laws, relatives or TBAs. Female and male respondents stated skipping meals were common. Nearly three quarters of females stated it was common for them to skip meals when they were breastfeeding. Part of this is traditional in that there is an expectation that *“visitors and males are first to be served food”.* (CM1; Additional File IV -Table S4).

**Table 4:** Nutrition practices among 859 Households in Jonglei South Sudan, June–July 2023

	N	All	IDPs	Returnees	Host Community	*p*-Value^a^	*p*-Value^a^
		Weighted % (95% CI)	Weighted % (95% CI)	Weighted % (95% CI)	Weighted % (95% CI)	IDP vs Host Community	Returnee vs Host Community
*Nutrition Practices*							
Breastfed last baby	543	94.9 (92.5–96.5)	96.9 (92.9–98.7)	93.8 (86.8–97.2)	93.7 (89.5–96.3)	0.154	0.969
Most common reasons for not breastfeeding^b^	28						
Difficulty breastfeeding		22.6 (10.0–43.3)	51.6 (16.3–85.4)	30.9 (7.3–71.7)	7.3 (1.0–37.9)	0.007	0.107
Mother had a medical problem		22.8 (10.5–42.6)	37.9 (9.5–78.1)	33.5 (8.2–73.8)	12.0 (3.0–37.8)	0.574	0.290
Baby had a medical problem		13.6 (4.8–32.9)	0.0 (0.0–0.0)	17.8 (2.4–65.6)	17.2 (5.0–45.3)	0.182	0.602
Milk did not come in		13.6 (4.8–33.0)	5.3 (0.6–32.6)	0.0 (0.0–0.0)	23.0 (7.6–52.1)	0.912	0.193
Baby would not latch		8.0 (2.0–27.0)	0.0 (0.0–0.0)	0.0 (0.0–0.0)	14.7 (3.6–43.8)	0.263	0.254
Immediate breastfeeding (within 1 h)	512	94.3 (91.7–96.2)	92.8 (87.6–96.0)	91.4 (83.0–95.9)	96.9 (93.4–98.6)	0.266	0.053
Average time (months) after birth infant given supplemental food/water (Range 0–12)	238	5.2 (4.8–5.6)	5.3 (4.6–5.9)	4.6 (3.7–5.6)	5.4 (4.9–6.0)	0.437	0.331
Exclusive breastfeeding for 6 months	238	26.0 (20.4–32.6)	19.4 (10.8–32.3)	25.3 (14.7–40.0)	30.2 (21.9–40.1)	0.019	0.402
Supplements given during first six months	242						
Cow’s milk		84.5 (78.9–88.8)	72.7 (59.5–82.8)	80.6 (66.5–89.7)	93.1 (87.1–96.4)	< 0.001	0.117
Water		70.3 (63.7–76.2)	69.3 (56.3–79.8)	67.7 (52.7–79.8)	72.1 (62.6–80.0)	0.446	0.807
Sorghum/rice porridge		29.0 (23.2–35.7)	49.0 (36.4–61.7)	40.1 (26.9–54.9)	12.3 (7.2–20.3)	< 0.001	< 0.001
Goat’s milk		15.0 (10.7–20.7)	18.9 (10.6–31.4)	19.6 (10.4–33.8)	10.7 (5.9–18.5)	0.202	0.098
Gave powdered milk instead of or in addition to breastmilk	535	17.0 (14.0–20.5)	14.6 (10.2–20.5)	30.7 (22.2–40.6)	12.6 (9.0–17.5)	0.141	< 0.001
Breastfeeding help in the community	586					0.637	0.037
Yes		78.3 (74.5–81.7)	80.9 (74.6–86.0)	84.5 (75.7–90.5)	73.7 (67.6–79.0)		
No		21.7 (18.3–25.5)	19.1 (14.0–25.4)	15.5 (9.5–24.3)	26.3 (21.0–32.4)		
Breastfeeding advice from	586						
Mother/Mother-in-law		44.7 (40.5–49.0)	40.7 (33.8–48.0)	59.9 (49.8–69.1)	41.3 (35.2–47.7)	0.532	0.002
Relative		40.5 (36.3–44.8)	49.1 (41.9–56.4)	47.3 (37.6–57.1)	30.8 (25.3–37.0)	< 0.001	< 0.001
TBA		20.6 (17.3–24.3)	24.4 (18.7–31.2)	16.1 (10.0–24.7)	19.5 (15.0–25.0)	0.694	0.053
Nurse midwife		5.9 (4.2–8.2)	4.2 (2.1–8.3)	4.6 (1.9–10.8)	7.7 (4.9–11.8)	0.049	0.250
Skipped meals when breastfeeding last child	484	72.4 (68.0–76.4)	64.6 (56.9–71.6)	76.3 (65.8–84.4)	77.4 (71.0–82.8)	0.004	0.991
Wife skipped meals when breastfeeding last child	212	73.5 (66.7–79.4)	79.9 (69.0–87.7)	76.3 (59.8–87.5)	67.2 (56.2–76.5)	0.319	0.359
Child screened for malnutrition^c^	736	40.3 (36.6–44.0)	43.2 (37.0–49.6)	42.1 (33.7–51.1)	37.3 (32.0–42.8)	0.138	0.331
Child treated for malnutrition^c^	725	26.7 (23.4–30.2)	28.0 (22.6–34.2)	26.1 (19.1–34.7)	25.9 (21.3–31.1)	0.568	0.960

*CI* confidence interval

^a^Categorical variables: Pearson ^2^ test; Continuous variables: two tailed t-test for means

^b^Percentages are calculated out of women who reported not breastfeeding their last child

^c^Percentages are calculated out of respondents with children under 5 years old in their household

^d^Other water sources include: lake/pond, piped into dwelling or yard/plot, protected spring or well, public tab/standpipe, rain water catchment

^e^Based on observation of data collector

#### Malnutrition screening and treatment practices

Forty percent of respondents reported they had a child screened for malnutrition. (Table 4) Based on qualitative interviews, and across ethnic groups, the best food is given to the man, especially protein, which is at the expense of women and girls. (DM1,3; Additional File IV -Table S4) However, boys are also subject to negative feeding practices. (DM1,3; Additional File IV -Table S4) Meals are withheld from boys aged 10–13 because they believe they will become lazy. (DM1,3, HM1; Additional File IV -Table S4) *“This is why there are many pre-adolescent and adolescent boys fishing, so they have food during their adolescent years.”* (DM1,3; Additional File IV -Table S4) At 14 years, boys are considered a man and feeding at home resumes where they are prioritized for the best food. (HC1; Additional File IV -Table S4) “*Girls [aged 10–13] are fed well because they are being marketed for marriage and looking malnourished does not bring a good bride price*.” (DM1; Additional File IV -Table S4) More than a quarter of respondents (26.7%) stated they had a child treated for malnutrition. Therapeutic foods distributed to children who are diagnosed with malnutrition frequently end up shared with the entire family. The malnourished child therefore may not get the treatment as prescribed and *“a diagnosis of malnutrition of one child allows the entire family to benefit nutritionally.”* (DM3; Additional File IV -Table S4).

### WASH practices

Only 40% of respondents stated there was enough water for washing, bathing, and drinking for everyone in the household. (Table 5) Estimated household water usage ranged from 7.9 to 10.1 L/person/day. Both males and females stated they fetch water roughly three times a day with 94% of females fetching water while they were pregnant. Both returnees (22.7%) and IDPs (19%) had a higher prevalence of pit latrines and hand washing stations (IDPs–12.6%; Returnees–17.6%; Host community–14%) when compared to the host community (13.1%). Soap was present in nearly a quarter of all households. Boreholes were more prevalent in host communities (37.9%), with IDPs (23.1%) having the least access to boreholes (*p* < 0.001). Sixty-two percent of all households listed unprotected water sources as their main source of water. For water storage, IDPs and returnees lacked covered buckets but had a higher proportion of households with covered jerry cans when compared to the host community. Only 15% of containers were free of dirt inside and outside and free of sediment or foreign objects. However, IDPs had cleaner water storage containers when compared to the host community (*p* = 0.02). Most households used water purification tablets. Three months prior to the survey, only a quarter of water collection sources were repaired or cleaned. A fifth of households stated there were water user committees in their area. Of the water user committees present, 56% were trained, 87% were active and 54% had a water safety plan.

**Table 5:** WASH practices among 859 Households in Jonglei South Sudan, June-July 2023

	N	All	IDPs	Returnees	Host Community	*p*-Value^a^	*p*-Value^a^
		Weighted % (95% CI)	Weighted % (95% CI)	Weighted % (95% CI)	Weighted % (95% CI)	IDP vs Host Community	Returnee vs Host Community
WASH Practices							
Agreement there is enough water for washing, bathing, drinking for everyone in the household	848	40.1 (36.6–43.6)	38.6 (32.9–44.6)	35.3 (27.9–43.5)	43.1 (38.0–48.3)	0.233	0.097
Estimated household water usage-liters/person/day	792	8.4 (7.8–8.9)	7.9 (7.1–8.7)	10.1 (8.1–12.1)	7.9 (7.5–8.4)	0.968	0.032
Fetches water							
Female	584	94.9 (92.6–96.5)	94.2 (89.5–96.9)	96.8 (90.3–99.0)	94.6 (91.0–96.8)	0.402	0.194
Male	269	36.9 (31.1–43.2)	52.9 (41.9–63.7)	49.9 (35.1–64.7)	21.5 (14.9–30.2)	< 0.001	< 0.001
Fetches water (No. of times/day)							
Female	550	3.2 (3.1–3.3)	3.2 (3.0–3.5)	3.1 (2.8–3.4)	3.3 (3.1–3.5)	0.812	0.320
Male	106	2.9 (2.6–3.2)	2.9 (2.4–3.4)	3.1 (2.6–3.6)	2.7 (2.1–3.3)	0.558	0.484
Distance to fetch water							
Minutes	657	37.5 (33.4–41.6)	42.6 (35.2–49.9)	40.6 (31.2–50.0)	31.5 (26.0–37.1)	0.011	0.092
Estimated Km	657	1.4 (1.1–1.7)	1.8 (1.2–2.4)	1.6 (0.9–2.4)	0.9 (0.5–1.4)		
Fetched water while pregnant	532	93.6 (91.0–95.4)	92.9 (88.0–95.9)	93.1 (86.1–96.8)	94.3 (90.4–96.6)	0.894	0.971
WASH provisions							
Pit latrine	836	17.0 (14.5–19.8)	19.0 (14.7–24.2)	22.7 (16.5–30.3)	13.1 (9.9–17.0)	0.034	0.006
Hand washing station	803	12.6 (10.4–15.2)	17.6 (13.5–22.6)	14.0 (9.0–21.2)	8.4 (5.9–11.8)	< 0.001	0.061
Soap	823	23.7 (20.8–26.9)	23.3 (18.7–28.7)	20.7 (14.7–28.2)	25.3 (20.9–30.2)	0.555	0.260
Main source of water	858					< 0.001	0.016
Borehole		32.8 (29.5–36.2)	23.1 (18.5–28.4)	37.4 (29.9–45.7)	38.0 (33.1–43.2)		
Unprotected spring		34.0 (30.7–37.4)	39.0 (33.2–45.0)	41.9 (34.1–50.1)	27.0 (22.6–31.9)		
River/stream		19.0 (16.4–21.9)	20.6 (16.2–26.0)	9.2 (5.4–15.2)	21.8 (17.8–26.4)		
Unprotected well		9.5 (7.5–11.8)	11.8 (8.4–16.4)	8.5 (4.9–14.4)	8.1 (5.6–11.5)		
Other^d^		4.8 (3.6–6.6)	5.5 (3.3–8.9)	3.0 (1.3–7.1)	5.1 (3.3–7.9)		
Main source of water for all cooking, drinking and hygiene	858					< 0.001	0.008
Borehole		32.8 (29.5–36.2)	23.1 (18.5–28.4)	37.6 (30.1–45.9)	37.9 (33.0–43.1)		
Unprotected spring		33.4 (30.1–36.8)	39.4 (33.6–45.5)	41.0 (33.2–49.2)	25.9 (21.6–30.7)		
River/stream		18.9 (16.3–21.8)	20.2 (15.8–25.5)	9.7 (5.8–15.9)	21.7 (17.8–26.2)		
Unprotected well		9.4 (7.5–11.8)	11.5 (8.1–16.0)	7.8 (4.3–13.6)	8.6 (6.1–12.1)		
Other^d^		5.5 (4.1–7.3)	5.8 (3.6–9.4)	3.9 (1.8–8.5)	5.9 (3.9–8.8)		
Water storage	859						
Bucket, covered		18.6 (16.0–21.5)	10.6 (7.4–14.8)	14.3 (9.4–21.1)	26.3 (22.0–31.0)	< 0.001	0.003
Bucket, uncovered		31.8 (28.6–35.2)	38.4 (32.7–44.5)	28.0 (21.3–36.0)	28.4 (24.0–33.3)	0.005	0.934
Jerry can, covered		31.7 (28.5–35.0)	32.8 (27.4–38.6)	38.7 (31.1–46.9)	28.0 (23.6–32.9)	0.170	0.014
Jerry can, uncovered		43.0 (39.5–46.5)	49.6 (43.6–55.6)	54.9 (46.7–62.9)	33.2 (28.5–38.3)	< 0.001	< 0.001
Pot, covered		19.1 (16.5–22.0)	15.3 (11.5–20.1)	27.0 (20.4–34.8)	18.7 (15.0–23.1)	0.238	0.031
Pot, uncovered		12.6 (10.4–15.1)	9.1 (6.2–13.4)	9.8 (5.9–15.8)	16.2 (12.7–20.5)	0.006	0.052
Clean water container^e^	811	14.8 (12.5–17.4)	18.2 (14.2–23.1)	15.4 (10.4–22.1)	11.9 (8.9–15.8)	0.022	0.292
Use of water purification tables	169						
Sodium Dichloroisocyanurate		47.3 (38.5–56.3)	47.3 (34.6–60.5)	70.3 (49.7–85.1)	35.0 (22.0–50.6)	0.215	0.006
Sodium hypochlorite		8.4 (4.5–14.9)	3.5 (0.9–12.8)	5.0 (0.7–28.3)	15.8 (7.6–30.0)	0.035	0.196
Combined flocculant/disinfectant		0.3 (0.0–1.9)	0.6 (0.1–4.3)	0.0 (0.0–0.0)	0.0 (0.0–0.0)	0.597	1.000
Chlorine		63.5 (54.4–71.6)	66.4 (52.6–77.9)	56.5 (36.6–74.5)	63.8 (48.2–76.9)	0.785	0.565
Water collection source repaired/cleaned in last 3 months	719	26.0 (22.8–29.5)	25.8 (20.7–31.6)	28.2 (20.4–37.5)	25.4 (20.8–30.5)	0.900	0.560
Water user committee present	698	19.4 (16.5–22.6)	20.4 (15.9–25.7)	15.9 (10.2–24.0)	19.9 (15.7–25.0)	0.891	0.349
Water user committee member							
Female	544	5.6 (3.9–7.8)	8.5 (5.4–13.2)	6.1 (2.8–13.2)	2.9 (1.3–6.2)	0.001	0.098
Male	256	8.2 (5.3–12.4)	10.5 (5.6–18.9)	2.4 (0.3–15.8)	8.7 (4.6–15.9)	0.746	0.230
Water use committee has:							
Training	178	56.1 (47.1–64.7)	81.6 (68.3–90.1)	24.6 (10.3–48.2)	41.8 (29.2–55.5)	< 0.001	0.198
Active	178	87.0 (79.6–92.0)	85.7 (73.8–92.7)	79.1 (51.6–93.1)	90.8 (78.6–96.4)	0.405	0.203
Water safety plan	181	53.8 (45.0–62.4)	81.9 (68.9–90.3)	23.3 (9.7–46.1)	36.9 (25.3–50.3)	< 0.001	0.282

*CI* confidence interval

^a^Categorical variables: Pearson ^2^ test; Continuous variables: two tailed *t*-test for means

^b^Percentages are calculated out of women who reported not breastfeeding their last child

^c^Percentages are calculated out of respondents with children under 5 years old in their household

^d^Other water sources include: lake/pond, piped into dwelling or yard/plot, protected spring or well, public tab/standpipe, rain water catchment

## Discussion

An overall lack of basic nutrition and WASH knowledge, negative attitudes and norms and poor practices among participants in the areas surveyed create significant barriers to decreasing morbidity and mortality especially among reproductive age women, infants, and children. Prior documented poor nutrition and WASH indicators do not reflect the current health situation of Jonglei state given the influx of IDPs and returnees [10, 18]. 

### Nutrition

The inability to identify three or more strategies to prevent starvation or malnutrition impacts improved nutrition practices. With low exclusive breastfeeding rates and use of prelacteal supplements and powdered milk supplements, it is not a surprise that nearly three-quarters of children were reported to be either screened or treated for malnutrition. Traditional beliefs in the survey area have an impact on nutrition practices including malnutrition among infants and children and maternal supplementation. The prohibition, based on traditional beliefs, around eating protein sources like ground nuts, chicken, and eggs limits available protein sources to prevent malnutrition and pregnancy complications such as anemia and low birth weight infants. Given the number of children screened and or under treatment for malnutrition, the lack of exclusive breastfeeding and poor complimentary feeding practices in the survey area suggests community mobilization around the importance of exclusive breastfeeding, using available proteins for complimentary feeding is warranted.

Promoting the need for an extra meal during pregnancy may not be realistic in South Sudan and especially among the areas surveyed where nearly three-quarters of pregnant females admitted to skipping meals while breastfeeding. Approximately 40% of households in South Sudan (~ 60% in this study) depend on agriculture and livestock herding as their main sources of income [10]. Many households rely on unsustainable income sources (e.g. charcoal making, collecting of firewood, grass, water and wild foods) that are traditionally used only in times of economic distress to complement farming and livestock rearing with a third using these unsustainable sources to generate household income [10]. The proportion of households relying on unsustainable income sources is especially high in Jonglei [10]. More than a third of families in Jonglei use markets as their main source of food supply and they spend 76% of total household cash on food [25]. Even with supply of supplemental fortified food and supplements, tradition, and culture may prohibit the ability of pregnant females to accept extra meals.

Sixteen percent of neonatal deaths could be averted if all infants were breastfed exclusively from the day of their birth and 22% averted if breastfeeding started within the first hour [26]. Early initiation of breastfeeding is defined as ‘provision of mothers’ breast milk to infants within the first hour of birth and ensures that the newborn receives colostrum which includes nutrients and antibodies that act as the infants first immunization and protection against diarrhea and neonatal sepsis until immunizations can be given [27]. Between 80 and 93% of respondents stated they breastfed their last baby and more than 93% of respondents stated they practiced early breastfeeding (within 1 h after birth) with 85–90% agreeing that early breastmilk protects babies against infections suggesting community mobilization and messaging may be successful. The most common reasons for non-breastfeeding were difficulty breastfeeding and if the mother had a medical problem. When asked if there was breastfeeding help in the community, 82% of respondents stated there was such help and that breastfeeding advice came from mothers-in-law and TBAs. BHWs and CHWs were not listed suggesting the Maternal, Infant and Young Child Nutrition Guidelines may not be implemented in the survey area [28]. 

Exclusive breastfeeding rates in this study (26%) are problematic. Compared with national rates, the rates of exclusive breastfeeding (≥ 6 months) are low in the areas surveyed (National–60%; this study 19.4% IDPs, 25.3% returnees, 30.2% host community) despite 80–90% of respondents agreeing that babies should be exclusively breastfed [10]. Our numbers are less than previously documented rates in Jonglei of 44% [10]. A variety of supplements were used for infants less than 6 months; cow’s milk or water being the most common. The rates of knowing that babies should be exclusively breastfed and the disconnect between exclusively for 6 months and the use of powdered milk and breastmilk substitutes among up to 30% of respondents is a worrying trend especially given the lack of clean water in the areas surveyed. Powdered milk is not the same as infant formula and does not supply the necessary nutrients that breastfeeding does [29]. Traditional practice of giving pre-lacteal supplements, cow’s milk, water, sorghum, or rice porridge, early breastfeeding cessation due to perceived insufficient milk supply or myths regarding babies being thirsty as instructed by mothers-in-laws and TBAs have an impact on exclusive breastfeeding rates. Basic education on breastfeeding, dispelling myths, and decreasing the use of powdered milk and breast milk substitutes is necessary.

Other important vitamin supplementation and micronutrient supplements should be considered in these areas given the food insecurity in these areas and the numbers of pregnant women skipping meals. South Sudan urges all pregnant and menstruating women and adolescent girls to receive 30–60 mg of elemental iron and 400 μg folate supplementation, especially for those living in settings where anemia is highly prevalent using CHWs and boma health workers (BHWs) to meet these goals [30]. World Food Program, supplying food within the survey areas, fortifies the food with iron (1.5 mg/d) and folic acid (300 μg/d) given the high levels of food insecurity in Jonglei [31]. 

Population data on micronutrient deficiencies is scant for South Sudan. Evidence of low dietary diversity among South Sudanese adds to micronutrient deficiencies [10]. In South Sudan, 16% of households are extremely food insecure: surviving on primarily cereals and consuming little proteins, vegetables and dairy products; 21% consume cereals supplemented with small and infrequent quantities of proteins (beef, chicken and or ground nuts), vegetables, sugar and oils and can be considered as moderate food insecure [10]. Micronutrient deficiencies are pervasive among females of reproductive age and children 6–59 months of age, especially during emergencies [30]. Those who are pregnant and lactating have an increased need for micronutrients [30]. Providing them with foods fortified with micronutrients is only one way to meet the recommended daily intake, however these foods may not meet fully the needs of certain nutritionally vulnerable subgroups such as those who are pregnant and lactating, or young children and require a daily multiple micronutrient supplement.

Globally, the most reported micronutrient deficiencies are vitamin A, iodine, and anemia/iron deficiency [32, 33]. Vitamin A deficiency (VAD) is a moderate to severe health problem among pregnant and lactating mothers to include increases in puerperal sepsis and possibly maternal mortality [33]. Vitamin A supplementation (VAS) among reproductive age, pregnant and lactating individuals appears to prevent the incidence of puerperal sepsis and milder postpartum febrile episodes and other morbidities [33]. 

Although infants who are exclusively breastfed to age 6 months are generally protected against VAD, in Jonglei, few reproductive age, lactating, and pregnant individuals receive VAS postpartum, and maternal nutrition and Vitamin A intake are poor so their breastmilk retinol may be low [34]. Since the exclusive breastfeeding rate is low (26%), increasing a mother’s breastmilk retinol would have positive effects for both mother and child, thus additional promotional efforts should be made to improve that and all the above practices to better ensure adequate micronutrient status and decreases mortality among children at age 6 months. [35, 36] Providing high-dose vitamin A capsules to children aged 6–59 months living where vitamin A is deficient increases their chance of survival by reducing all-cause mortality by 23–24%, measles morbidity and mortality by 50%, diarrheal disease mortality by 28–33%, and incidence of diarrhea by 15%. [35, 37, 38], Since Jonglei has lower coverage of VAS than the national average, VAS is cost-effective and an important means to decrease morbidity and mortality [39]. 

### WASH

With nearly 62% of all respondents able to identify three reasons for handwashing and positive attitudes about WASH topics and practices suggests messaging in communities has created some behavior change. However, knowledge may not be able to overcome the lack of clean water for washing, bathing, and drinking for everyone in the household, especially for returnees and IDPs where there are boreholes for the host community leaving returnees and IDPs to use unprotected water sources. (Additional File 3; Figure S1).

Although most households could identify reasons for handwashing only 40% of household respondents could name three warning signs that a child with diarrhea should be seen by skilled providers. Healthy WASH practices were low even with agreement on statements that showed knowledge of healthy WASH attitudes. Washing during post-partum bleeding alleviates the risk of infection therefore not washing for seven days in addition to sitting in sand can lead to unnecessary infection. Data from this survey shows largely unimproved water sources with fewer than 25% of respondents having pit latrines, handwashing stations, and/or soap. Handwashing with soap is shown to reduce diarrhea risk by 31% and acute respiratory infection risk by 21% and is more effective than with water alone [40–42]. Having soap in the household does not ensure handwashing includes soap and water use for hand hygiene. [43] The lower odds of handwashing knowledge in households with pit latrines and hand washing stations suggests hygiene promotion and education is necessary periodically to reduce diarrheal disease and to reinforce the need for handwashing with soap and water even when WASH provisions are available [41, 42, 44]. 

Only two percent of South Sudanese households have water on the premises [10]. Previous data showed that 78% of households in Jonglei have access to improved drinking water sources, whereas our study found that only 33% of households in the survey area had access to improved drinking water sources [10]. Safe water for IDPs was less prevalent and given that these areas are flooded for more than half the year and only a quarter of these sources are cleaned and/or improved, it is likely the boreholes may not guarantee safe water. Household water treatment is effective, simple, and inexpensive which is being practiced in the survey areas [45]. Borehole platform installations resistant to flooding are necessary to improve water sources in addition to new boreholes in the IDP areas to reduce the time it takes to fetch water [46].

A minimum of 7.5 L per capita per day will meet the requirements of most people under most conditions [45, 47]. Our study shows this minimum is met by all households, however the accepted 20 L per capita should be assured to take care of basic hygiene needs and basic food hygiene in addition to the need for covered storage containers [47]. 

Previous studies in Jonglei showed that only 39% percent of children with diarrhea received oral rehydration solution (down from 64% in 2006) with differences based on household wealth where higher income households used ORS compared to lower income households [10]. Children in urban households were also more likely to receive ORS (57%) when compared to rural households (50%) [10]. Ten percent of mothers did not orally replete fluids and 11% stopped feeding the child [10]. Given the lack of education especially among females and the inability of the survey population to read the directions on ORS packs, it is not surprising that ORS usage is low. In South Sudan, co-packaged ORS and zinc is not available and zinc coverage is only 2% [10, 48]. Zinc helps with the absorption of water and electrolytes, improves regeneration of the intestinal epithelium, improves the levels of brush border enzymes, and enhances the immune system in addition to serval other physiologic processes to inhibit fluid loss [49]. Zinc plays an important role in recovery and reducing the morbidity and mortality associated with diarrhea especially among malnourished children [49]. Supplemental zinc results in a shorter duration of diarrhea, reduces the number of stools and stool output, reduces the risk of persistent diarrhea, and may reduce the risk of subsequent illness and increase weight gain [50]. The estimated prevalence of severe malnutrition in Jonglei was 14% in 2010. This may be higher due to displacement and poor water sources in the areas surveyed. New data suggests that lower doses of zinc (5 mg or 10 mg per day) in children with acute diarrhea had diarrhea outcomes similar to those in children receiving 20 mg but had less vomiting [51]. Given the lack of co-packaging of zinc and ORS, non-use of ORS, and malnutrition rates across the survey area, increasing ORS and zinc use and making sure instructions are infographics will be an important step in reducing child morbidity and mortality.

## Limitations

The weighted findings of this study represent the adult populations in the ten bomas in Ayod, Pigi, Nyirol, and Fangak counties surveyed and cannot be generalized to populations in other bomas, counties or among children. The sample weights are based on population estimates from the Republic of South Sudan National Bureau of Statistics, United Nations estimates and DHIS2, and may introduce bias given there is no current census data to rely on with the last census taking place in 2008. The design of this study allows for the determination of population characteristics but not causality between those characteristics and outcomes of interest. As Dinka and Neur are primarily oral languages, the supervisors were more comfortable using instruments written in English and then translated to Dinka and Neur verbally with qualitative responses written in English. It is possible there is a loss of detail from translation and back-translation. However, our supervisors were experienced in doing qualitative surveys in this manner and qualitative interviews were used for triangulation and not as an in-depth qualitative study. Qualitative interviews represent those interviewed and cannot be generalized to the wider population. Although social desirability bias is possible it is limited in this anonymous survey and because respondents were interviewed alone. Furthermore, nutrition attitudes are less of a sensitive topic than something like substance abuse and attitudes were determined were through different questioning techniques (“in your opinion” and agree/disagree) and with options to not answer or say, “I don’t know”. Interviewers were careful to explain there would be no material gain by participation in the study; however, respondents may have underestimated or exaggerated responses if they thought it would be in their interest to do so.

## Conclusions

Given the link between nutrition and WASH program, nutrition programs must improve WASH concurently [6, 7]. Evidence based interventions should be the priority and include, at a minimum, micronutrient supplementation and or food fortification with vitamin A, iron, folic acid, and iodine with a focus on ensuring those who are reproductive age, pregnant or lactating have adequate supplemental food [16, 17, 38, 41]. Immediate and exclusive breastfeeding messaging and addressing negative norms should be a priority to decrease infant mortality, with a focus on exclusive breastfeeding given the poor rates in the survey area [16, 17, 26]. Improved and flood resistant sources for adequate amounts of clean water, clean covered water storage container distribution and use, ORS and zinc distribution and education are necessary to decrease diarrheal rates among all households whether they are IDP, returnees or the host community [52]. 

## Supplementary Information

Additional file 1.

Additional file 2.

Additional file 3.

Additional file 4.

Additional file 5.

## Data Availability

No datasets were generated or analysed during the current study.

## Abbreviations

BHW: Boma health worker
CHW: Community health worker
DHIS2: District health information system 2
EPI: Expanded program on immunization
IDP: Internally displaced person
KAP: Knowledge, attitude and practice
MOH: Ministry of health
NGO: Non-governmental organization
ORS: Oral rehydration solution
TBA: Traditional birth attendant
U5MR: Under-five mortality rate
VAD: Vitamin A deficiency
VAS: Vitamin A supplementation
WASH: Water, sanitation and hygiene
WHO: World health organization
