# Supplementary material for: A mixed-methods nutrition, water, sanitation and hygiene knowledge, attitudes, and practices survey of IDPs, returnees, and host community members in four counties of Jonglei state, South Sudan
Source: J Health Popul Nutr. 2025 Feb 25;44:48. doi: 10.1186/s41043-025-00789-3 (PMC11863811; doi:10.1186/s41043-025-00789-3)
Supplement: Supplementary file 1 — Additional file 1. [file 41043_2025_789_MOESM1_ESM.pdf]

# JESI Baseline 2023

**1. Enter a date and time**

yyyy-mm-dd hh:mm

**2. Data Collector ID**

- 001
- 002
- 003
- 004
- 005
- 006
- 007
- 008
- 009
- 010
- 011
- 012
- Ann
- David
- Francis
- Lazarus

**2a. Interview ID**

**3. Is this interview of an IDP, a returnee or a member of the host community?**

- Internally Displaced Person (South Sudanese but not from the area of interview)
- Returnee (South Sudanese who came back from elsewhere in the last 6 months)
- Host community member

**4. The sex of the respondent is?**

- Male
- Female

**5. County**

- Canal/Pigi
- Fangak
- Ayod
- Nyirol

**6a. Pigi/Canal Payam**

- Canal

**6b. Ayod Payam**

- Mogok

**6c. Nyirol Payam**

- Pultruk
- Guer

**6d. Fangak Payam**

- Pagwir
- Toch

**7. Mogok Boma**

- Magok-Panyang
- Gar
- Kurwai

**7a. Pultruk Boma**

- Bariak

**7b. Pading Boma**

- Guer

**7c. Canal Boma**

- Pigi
- Korfulus
- Mat

**7d. Pagwir Boma**

- Pagwir

**7e. Toch Boma**

- Tock

CONSENT STATEMENT

*Read the entire statement to the person you are interviewing*

---

We are working with IMA, a non-governmental organization from the United States. IMA has been working in South Sudan on many projects. IMA also works in other countries with the support of many governments, private donors and other international organizations to help them reach as many people as possible. However, they do not support any political or religious group.

---

We are conducting a survey to understand the health and needs of internally displaced persons, returnees and community members hosting the internally displaced persons in your area. We have asked permission from your community leaders to conduct this survey. We will read questions to you and ask for answers to each question. The findings of this study will be used to try to improve health in your community especially of pregnant women, babies and children. We are not here to provide humanitarian assistance at this time. Your participation is voluntary. Your answers may not directly benefit you, but they will help organizations and the government to plan services for you, your family, and your community. We realize that many people have suffered greatly and may have much to tell. But this survey requires only BRIEF responses to a limited number of questions. We expect this will take about 30 minutes of your time.

---

You do not need to give us your name. Your household was randomly selected among many other households. None of your answers will be shared with anyone else. It will not be possible to personally identify your answers. We do not feel that this survey puts you at risk, however they may be risk that we are unaware of. If we felt there was risk, we would not have approached your household at all.

---

Your participation in this study is voluntary. You may decide not to participate, or you may leave the study at any time. Your decision will not result in any penalty or loss of benefits to which you are otherwise entitled. Your alternative is not to participate in the survey.

---

You do not need to give us your name at all. None of your answers will be shared with anyone else and it will not be possible to know your answers from anyone else's answers. However, the researchers and the IRB referenced below will see your answers. Even if you do provide your name, it will not be kept with the answers to the survey questions to maintain your confidentiality. If you do not understand a question, please ask me to explain it to you. Please note that some questions that we ask are sensitive, therefore it is important that we have some privacy for our conversation. You are free to stop at any time during the interview. If a question makes you uncomfortable, we will skip the question and go to the next question. If you have any questions, concerns, or complaints about the survey, or if you feel that participating in the research has caused you harm, you can get more information from your interviewer or your community health worker in your area.

---

This research is being overseen by an Institutional Review Board ("IRB"). An IRB is a group of people who perform independent review of research studies. Your community health worker will be able to help you reach them if you have questions, concerns, or complaints that are not being answered by the research team, you are not getting answers from the research team, you cannot reach the research team, you want to talk to someone else about the research, or if you have questions about your rights as a research subject.

---

**8. May I ask you some questions?**

- Yes
- No

**9r. Why do you refuse to participate in the survey?**

- Lacks time
- Fears reprisal
- Tired of doing surveys
- Nothing has changed with these surveys
- Doesn't feel well
- No response
- Other

**9ra. Other reason -refusal to participate**

______________________________

**10r. Thank you for your time, we are sorry you are unwilling or unable to participate. I will respect your desire not to participate, can you please tell me how old you are before I leave? (years).**

*Enter "99" if they refuse to say their age*

______________________________

**END SURVEY**

*Data Collector: check "Go to end" and move to the next household*

hh:mm

**9. Do you know your age in years?**

- Yes
- No
- Prefer not to say

**9a. How old are you or how old do you think you are? (years)**

*MUST BE 18 years or older or if less than 18 they must be married: Enter- "99" if they refuse/don't know age*

______________________________

**10. How many years of formal education do you have? (of no formal education - enter "0")**

*(if they refuse to answer put "99")*

______________________________

**11. Do you work and contribute to the household income?**

- Yes
- No
- Prefer not to answer

**12. What is your occupation?**

- Not working
- Government work
- Small business
- Professional
- Student
- Farming
- Day labor
- Homemaker
- Other
- Prefer not to say

**13. Occupation - Specify other**

---

**14. Do you know much income your household has per month?**

- Yes
- No
- Don't know
- Prefer not to say

**14a. How much does your household have for income each month? (if none, please mark as "0")**

---

**14b. In the amount you gave me, what currency are you using?**

- South Sudanese pound
- US dollars
- Other

**14b. Currency Other**

*Example: maize, melawa (add currency value of maize or the cost of a melawa of maize etc.)*

---

**15. What is your marital status?**

- Never married
- Married
- With a partner
- Wife/husband missing due to conflict
- Divorced/separated/no longer in partnership
- Widow/Widower
- Prefer not to say

**16f. How many wives does your husband have? (enter "0" if never married; enter 99 if prefers not to say)**

______________________

**16a. What wife number are you? (enter "0" if never married;enter 99 if prefers not to say)**

______________________

**16m. How many wives do you have? (enter "0" if never married; enter "99" if prefers not to say)**

______________________

**17. At what age did you marry?**
*(enter "0" if does not know or never married or "99" if they prefer not to say)*

______________________

**18. Do you have a disability?**

- Yes
- No
- Don't know
- Prefer not to say

**18a. What type or types of disability do you have?**

- [ ] Vision
- [ ] Hearing
- [ ] Mobility
- [ ] Cognition (Remembering)
- [ ] Self-Care
- [ ] Communication
- [ ] None of the above
- [ ] Don't know

**19. How long have you been in this house? (write in a number only; enter "99" if they are don't know or prefer not to say)**

______________________________

**19a. Is the time in the house days, weeks, months or years (pick only one)**

- ○ Days
- ○ Weeks
- ○ Months
- ○ Years
- ○ Prefer not to say

**20. In the last 2 years, how many times have you been displaced or had to move for any reason?**

- ○ None
- ○ 1
- ○ 2
- ○ 3
- ○ 4
- ○ 5
- ○ Other
- ○ Prefer not to say

**20a. Other number**

______________________________

**21. Does your household own land?**

- ◯ Yes
- ◯ No
- ◯ Don't know
- ◯ Prefer not to say

**22. DATA COLLECTOR - observe the tukul - check all that apply**

- ☐ Mud brick house thatch roof
- ☐ Mud brick house no roof
- ☐ Plastic sheet
- ☐ Pit latrine
- ☐ Burn pit for garbage
- ☐ Prefer not to say/Skip

**23. What is your main source for water? (check only one)**

- ◯ Piped into dwelling
- ◯ Piped into yard/plot
- ◯ Public tap/standpipe
- ◯ Borehole
- ◯ Protected well
- ◯ Unprotected well
- ◯ Protected Spring
- ◯ Unprotected Spring
- ◯ River/stream
- ◯ Lake/pond
- ◯ Rain water catchment
- ◯ Tanker truck
- ◯ Cart with small tank/drum
- ◯ Bottled water
- ◯ Other
- ◯ Prefer not to say
- ◯ Don't know

**23a. Specify other water source**

___

**24. What water source do you use for ALL cooking, drinking and hygiene? (pick one major source)**

- Piped into dwelling
- Piped into yard/plot
- Public tap/standpipe
- Borehole
- Protected well
- Unprotected well
- Protected Spring
- Unprotected Spring
- River/stream
- Lake/pond
- Rain water catchment
- Tanker truck
- Cart with small tank/drum
- Bottled water
- Other (specify)
- Prefer not to say
- Don't know

**24a. Specify other water source used for ALL cooking, drinking and hygiene**

______________________________

**25. Is there enough water for bathing, washing hands, drinking for everyone in the household?**

- Yes
- No
- Don't know
- Prefer not to say

**26. How many people (including yourself) eat and sleep in this household?**

*Enter "0" if they are the only one in household; enter "99" if they prefer not to say*

______________________________

**27. How many liters of water does the household use per day?**

- 1 jerry can
- 2 jerry cans
- 3 jerry cans
- 4 jerry cans
- 5 jerry cans
- Other (specify) liters
- Don't know
- Prefer not to say

**27a. Other - specify liters of water used per day in the household**

---

**28. Do you fetch water?**

- Yes
- No
- Don't know
- Prefer not to say

**28a. Do you or did you fetch water when pregnant?**

- Yes
- No
- Have not been pregnant
- Don't know
- Prefer not to say

**29. How many times a day do you fetch water?**

- 1
- 2
- 3
- 4
- 5
- More than 5 times per day
- Prefer not to say

**30. How many minutes does it take to fetch water and then come back to the household?**

*Enter "0" if they do not know, enter "99" if they prefer not to say*

______________________________

**31. How many treated mosquito nets does your household have?**

*Enter "0" if they do not know, enter "99" if they prefer not to say*

______________________________

**32. Can you read?**

- ○ Yes
- ○ No
- ○ Don't know/Cannot remember
- ○ Prefer not to say
- ○ Yes, but visual disability (blind)

**33. DATA COLLECTOR: Ask respondent to read the directions on an ORS package.**

- ○ Could not read
- ○ Read with difficulty
- ○ Read easily
- ○ Refused to read

**34. Can you read numbers?**

- ○ Yes
- ○ No
- ○ Don't know/Cannot remember
- ○ Prefer not to say
- ○ Yes but visual disability (blind)

**35. DATA COLLECTOR: Ask respondent to point to the numbers 5, 8 and Zero.**

- ○ Could not point to any of the numbers
- ○ Pointed to only 1-2 of 3 numbers
- ○ Pointed to all 3 numbers
- ○ Refused

DATA COLLECTOR: Be sure there is no one else listening to the interview and you are alone with the respondent - if there are others, politely as them to leave so you can continue the survey.

______________________________

**36f. How many total pregnancies have you had? (include current pregnancy and miscarriages)**
*(If none, enter "0"; if they don't know, can't remember or prefers not to say enter "99")*

______________________

**36m. How many total pregnancies has your wife had? (include current pregnancy and miscarriages)**
*(If none, enter "0"; if they don't know, can't remember or prefers not to say enter "99")*

______________________

**37f. Were any of your pregnancies lost because of violence?**

- Yes
- No
- Prefer not to say
- Don't know/Don't remember

**37m. Were any of your wife's (wife in this household) pregnancies lost because of violence?**

- Yes
- No
- Don't know/Don't remember
- Prefer not to say

**38f. Are you pregnant now?**
*Read all answers: " You can say yes, no, not sure, I prefer not to say or don't know?"*

- Yes
- No
- Not sure
- Prefer not to say
- Don't know

**38m. Is your wife/partner (in this household) pregnant now?**

- Yes
- No
- Not sure
- Prefer not to say
- Don't know

**39f. Have you had circumcision?**

- Yes
- No
- Don't know/Don't remember
- Prefer not to say

**39fa. At what age were you circumcised?**

*Enter 99 if don't know; Enter 98 if prefers not to say*

______________________________

**39fb. Has the circumcision caused problems in childbirth?**

- Yes
- No
- Don't know/Don't remember
- Prefer not to say
- Have not had a pregnancy

**40. In your lifetime, have you ever experienced molestation, being forced to undress or stripped of clothing, forced intercourse and other sexual acts by anyone?**

- Yes
- No
- Don't know/Don't remember
- Prefer not to say

**40a. Did you seek care at a clinic after this happened to you?**

- Yes
- No, I did not seek care
- No, because there was no clinic available at that time
- Don't know/Don't remember
- Prefer not to say

**41. In your lifetime, have you been subjected to violence, such as beatings, by a spouse or partner?**

- Yes
- No
- Prefer not to say
- Don't know/Don't remember

**41a. Did you seek care at a clinic after this happened to you?**

- Yes
- No, I did not seek care
- No, because there was no clinic available at that time
- Don't know/Don't remember
- Prefer not to say

**42f. Are you using anything to prevent pregnancy?**

- Yes
- No
- Prefer not to say
- Don't know

**42fa. What method of family planning are you using to prevent pregnancy?**

- Oral contraceptives (pills)
- Male condoms
- Implant (in the arm)
- Intrauterine device (in the uterus)
- Other (specify)
- Prefer not to say

**42fb. Specify other method of family planning you are using to prevent pregnancy**

______________________________

**42m. Are you and your wife/partner (in this household) using anything to prevent pregnancy?**

- Yes
- No
- Don't know
- Prefer not to say

**42ma. What method of family planning are you using to prevent pregnancy?**

- Oral contraceptives (pills)
- Male condoms
- Implant (in the arm)
- Intrauterine device (in the uterus)
- Other (specify)
- Don't know
- Prefer not to say

**42b. Specify other method of family planning you are using to prevent pregnancy**

**43. Do you want to use anything to prevent pregnancy?**

- Yes
- No
- Don't know
- Prefer not to say

**44. What would stop you from using something to prevent pregnancy?**

- Nothing
- My husband/my wife
- Mother/Mother-in-law
- Not available
- Cost
- Not traditional
- Other (specify)
- Don't know
- Prefer not to say

**44a. Specify Other: what would stop you from using something to prevent pregnancy**

**44ma. Have you used condoms?**

- Yes
- No
- Don't know/Don't remember
- Prefer not to say

**44mb. Would you use condoms?**

- Yes
- No
- Don't know
- Prefer not to say

**44mc. Why won't you use condoms?**

Please check to make sure no one else is listening - politely ask others to leave so you can continue the interview in private.

**45. Do you have children less than 5 years of age in this household?**

- Yes
- No
- Don't know/Don't remember
- Prefer not to say

**45a. How many children in your household are under 5 years old?**
*Enter "99" if prefer not to say or don't know*

**45b. How many children in your household are under 2 years old?**
*Enter "99" if prefer not to say or don't know*

**46f. In your view, what should you do (medically) when you are pregnant?**
*(Do not read; check all listed)*

- [ ] Could not list anything
- [ ] See a healthcare provider
- [ ] Eat well
- [ ] Sleep under a bed net
- [ ] Get weighed
- [ ] Get immunizations such as tetanus toxoid
- [ ] Have blood pressure measured
- [ ] Receive iron tablets
- [ ] Give a blood sample
- [ ] Give a urine sample
- [ ] Nothing
- [ ] Don't know/Don't remember
- [ ] Other
- [ ] Prefer not to say

**46fa.Other: specify other things that should be done when you are pregnant**

________________________________________

**46m. In your view, what should your wife do (medically) when she is pregnant?**
*(Do not read; check all listed)*

- [ ] See a healthcare provider
- [ ] Eat well
- [ ] Sleep under a bed net
- [ ] Get weighed
- [ ] Get immunizations such as tetanus toxoid
- [ ] Have blood pressure measured
- [ ] Receive iron tablets
- [ ] Give a blood sample
- [ ] Give a urine sample
- [ ] Nothing
- [ ] Don't know/Don't remember
- [ ] Could not list anything
- [ ] Other
- [ ] Prefer not to say

**46ma. Other: specify things that should be done when your wife is pregnant**

---

**47f. During your last or current pregnancy did you have any antenatal checks?**

*Antenatal care means at a visit any of the following was done: Blood pressure measurement, Urine testing, Blood testing, Weight/height measurement*

- Yes
- No
- Don't know/Don't remember
- Prefer not to say

**47fa. How many antenatal checks did you have with your last pregnancy?**

*Enter "99" if they prefer not say*

---

**47fb. How many months pregnant were you when you had your first check on your last or current pregnancy?**

*Enter "99" if they prefer not to say*

---

**47fc. Who did the pregnancy check?**

- Medical Doctor
- Nurse midwife
- Midwife
- Traditional Birth Attendant
- Community health worker
- Relative
- Neighbor
- Don't know/remember
- Other (specify)
- Prefer not to say

**47fco. Specify other: (who did the pregnancy check?)**

---

**47fd. Why did you not have any health checks for your last or current pregnant?**

*(Check the main reason)*

- [ ] It was not needed
- [ ] Not available (no clinic)
- [ ] Clinic too far away
- [ ] Cost Not allowed to by husband
- [ ] Family members did not allow
- [ ] Too busy
- [ ] Had to care for kids
- [ ] Too much work
- [ ] Don't know
- [ ] Other
- [ ] Prefer not to say

**47fdo. Specify other reason you did not have any health checks for your last or current pregnancy**

______________________________

**47m. During your wife/partner's (in this household) last or current pregnancy did she have any antenatal checks?**

- ○ Yes
- ○ No
- ○ Don't know/Don't remember
- ○ Prefer not to say

**47ma. How many antenatal checks did your wife/partner (in this household) have with her last or current pregnancy?**

*Enter "98" if they prefer not to say*

______________________________

**47mb. How many months pregnant was she when she had your first check on her last or current pregnancy?**

*Enter "99" if they do not know; Enter "98" if they prefer not to say*

______________________________

**47mc. Who did the pregnancy check?**

- Medical Doctor
- Nurse midwife
- Midwife
- Traditional Birth Attendant
- Community health worker
- Relative
- Neighbor
- Don't know/remember
- Other (specify)
- Prefer not to say

**47mco. Specify other: (who did the pregnancy check?)**

---

**47md. Why did your wife (in this household) not have any health checks for her last or current pregnancy?**
*(Check the main reason)*

- It was not needed
- Not available (no clinic)
- Clinic too far away
- Cost Not allowed to by husband
- Family members did not allow
- Too busy
- Had to care for kids
- Too much work
- Don't know
- Other
- Prefer not to say

**Specify other reason your wife did not have any health checks for her last or current pregnancy**

---

**48. When should a pregnant woman immediately go or be taken to a health facility?**

*(Do not read; check all listed)*

- [ ] Vaginal bleeding
- [ ] Convulsions (fits)
- [ ] Loss of consciousness
- [ ] Severe headaches with or without dizziness
- [ ] Fever Persistent vomiting
- [ ] Severe Anemia
- [ ] Baby stops moving
- [ ] Early rupture of membranes (bleeding/water)
- [ ] Abortion
- [ ] Difficulty breathing (especially with dizziness and/or very pale skin)
- [ ] Contractions/labor pains, or bag of water breaking before 36 weeks gestation
- [ ] Weakness (Pale skin)
- [ ] Foul Smelling Vaginal Discharge
- [ ] Swelling of feet and hands
- [ ] Lower abdominal and pelvic pain
- [ ] Other
- [ ] Don't know/Don't remember
- [ ] Could not list any
- [ ] Prefer not to say

**48a.Specify other when a pregnant woman should immediately go to a health facility**

______________________________________________

**49. I am pleased you could name some of these signs, where did you learn about these signs?**

*(Check all listed; Do not read)*

- [ ] My wife had or I had these signs
- [ ] Clinic
- [ ] TBA
- [ ] CHW
- [ ] Relative
- [ ] Mother/mother-in-law
- [ ] Written material (newspaper, fliers, billboards)
- [ ] Radio
- [ ] Mobile messages
- [ ] TV
- [ ] Other
- [ ] Prefer not to say

**49a.Specify other person/place that you learned signs from**

______________________________

**50f. Who was with you during the birth of your last child?**

*(select all that apply)*

- [ ] Medical Doctor
- [ ] Midwife
- [ ] Nurse
- [ ] Community health worker
- [ ] TBA
- [ ] Family member
- [ ] Community member
- [ ] Delivered alone/no one
- [ ] Other
- [ ] Prefer not to say

**Specify other person with you during the birth of your last child**

______________________________

**50m. Who was with your wife (from this household) during the birth of your last child?**
*(select all that apply)*

- [ ] Doctor
- [ ] Midwife
- [ ] Nurse
- [ ] CHW
- [ ] TBA
- [ ] Family member
- [ ] Community member
- [ ] Delivered alone/no one
- [ ] Other
- [ ] Prefer not to say

**50ma. Specify other person with your wife (in this household) during the birth of your last child**

______________________________

**51f. Where did you deliver your last baby?**

- ( ) Clinic
- ( ) Hospital
- ( ) Home
- ( ) Other

**51fa. Specify other place you delivered your last baby**

______________________________

**51m. Where did your wife deliver your last baby?**

- ( ) Clinic
- ( ) Hospital
- ( ) Home
- ( ) Other
- ( ) Prefer not to say

**Specify other place you delivered your last baby**

______________________________

**52f. When your last baby was born, were you seen by anyone for health care just after the delivery?**

- Yes
- No
- Don't know/Don't remember
- Prefer not to say

**52m. When your last baby was born, was your wife (in this household) seen by anyone for health care just after the delivery?**

- Yes
- No
- Don't know/Don't remember
- Prefer not to say

**53f. Who saw you and your baby?**

- Medical Doctor
- Nurse midwife
- Midwife
- Traditional Birth Attendant
- Community health worker
- Relative
- Don't know/remember
- Other
- Prefer not to say

**53fa. Specify other person who saw you and your baby just after delivery**

______________________________

**53m. Who saw your wife (in this household) and your baby?**

- Medical Doctor
- Nurse midwife
- Midwife
- Traditional Birth Attendant
- Community health worker
- Relative
- Don't know/remember
- Other
- Prefer not to say

**Specify other person who saw your wife and your baby just after delivery**

---

**54f. How long after the delivery were you and your baby seen?**

- Within 6 hours
- 6-12 hours
- 12-24 hours
- 24-48 hours
- 48-72 hours
- Don't know/Don't remember
- Other
- Prefer not to say

**54fa. Specify other - when were you and your baby seen?**

---

**54m. How long after the delivery were you and your baby seen?**

- Within 6 hours
- 6-12 hours
- 12-24 hours
- 24-48 hours
- 48-72 hours
- Don't know/Don't remember
- Other

**54ma. Specify other - when were you and your baby seen?**

---

**55. Can you list some essentials to taking care of a newborn?**

*(Do not read; Check all listed)*

- [ ] Could not list any
- [ ] Breastfeeding
- [ ] Taking the baby for clinic visits
- [ ] Weighing the baby
- [ ] Vitamin A
- [ ] Immunizations
- [ ] Keeping the baby warm
- [ ] Care just after birth
- [ ] Knowing when the baby is sick
- [ ] Immediate and thorough drying
- [ ] Skin-to-skin contact
- [ ] Delayed bathing
- [ ] Cord clamping
- [ ] Clean cord care
- [ ] Don't know/Don't remember
- [ ] Other
- [ ] Prefer not to say

**55a. Specify other essentials of newborn care**

______________________________

**56. I am pleased you could name some of these essentials, where did you learn about these?**

*(Check all listed)*

- [ ] My wife/ my husband
- [ ] Clinic
- [ ] TBA
- [ ] CHW
- [ ] Relative
- [ ] Mother/mother-in-law
- [ ] Radio
- [ ] Written material (newspaper, fliers, billboards)
- [ ] Mobile messages
- [ ] TV
- [ ] Don't know/Don't remember
- [ ] Other
- [ ] Prefer not to say

**56a. Specify other essential(s)**

______________________________

**57. In your opinion, when should a newborn baby (up to one month old) be immediately taken to a health facility?**

*(Check all listed; Do not read)*

- [ ] Could not list any
- [ ] Fits/convulsions
- [ ] High fever
- [ ] Poor breastfeeding
- [ ] Refusing to drink milk
- [ ] Lethargic/unresponsive
- [ ] Difficulty breathing
- [ ] Baby is cold to the touch
- [ ] Baby is very small
- [ ] Baby is born early
- [ ] Swollen abdomen
- [ ] Yellow skin/palms/feet
- [ ] Redness/pus around umbilical cord
- [ ] White spots on mouth/tongue
- [ ] Vomiting or spitting up a lot
- [ ] Skin rash
- [ ] There is no reason for a newborn to be seen
- [ ] Don't know/Don't remember
- [ ] Other
- [ ] Prefer not to say

**57a. Specify other reasons a newborn should be immediately taken to a health facility**

______________________________________________

**58. I am pleased you could name some of these reasons, where did you learn about these?**

*(Check all listed; Do not read)*

- [ ] My wife/ my husband
- [ ] Clinic
- [ ] TBA
- [ ] CHW
- [ ] Relative
- [ ] Mother/mother-in-law
- [ ] Radio
- [ ] Written material (newspaper, fliers, billboards)
- [ ] TV
- [ ] Mobile messages
- [ ] Don't know/Don't remember
- [ ] Other
- [ ] Prefer not to say

**58a. Specify other reasons**

---

**59. If you thought your newborn needed to go to a clinic for care, what would stop you?**

- [ ] Nothing
- [ ] Newborns do not need to go to a clinic
- [ ] I need permission from my husband/partner
- [ ] I need permission from my mother/mother-in-law
- [ ] The cost
- [ ] No clinic
- [ ] Clinic is too far away
- [ ] Concerns about security
- [ ] I don't have or can't pay for transportation
- [ ] The clinic cannot help (no meds, no staff)
- [ ] Other
- [ ] I don't know
- [ ] Prefer not to say

**59a. Specify other reasons that would stop you for taking a newborn to a clinic**

---

**60f. Did you breastfeed your last baby?**

- ◯ Yes
- ◯ No
- ◯ Don't know/Don't remember
- ◯ Prefer not to say

**60m. Did your wife (in this household) breastfeed your last baby?**

- ◯ Yes
- ◯ No
- ◯ Don't know/Don't remember
- ◯ Prefer not to say

**61f. Why did you not breastfeed your last baby?**

*(Check all listed; Do not read)*

- ☐ Difficulty breastfeeding
- ☐ Advised against it
- ☐ Had a medical problem
- ☐ Baby had a medical problem
- ☐ Husband did not want mother to breastfeed
- ☐ Did not have time
- ☐ Milk did not come in
- ☐ Baby would not latch on
- ☐ Clinic wanted the baby on Plumpynut
- ☐ Preferred formula
- ☐ Other
- ☐ Prefer not to say

**61fa. Specify other reason you did not breastfeed**

_______________________________________________

**61m. Why did your wife (in this household) not breastfeed your last baby?**

*(Check all listed; Do not read)*

- [ ] Difficulty breastfeeding
- [ ] Advised against it
- [ ] Had a medical problem
- [ ] Baby had a medical problem
- [ ] Husband did not want mother to breastfeed
- [ ] Did not have time
- [ ] Milk did not come in
- [ ] Baby would not latch on
- [ ] Clinic wanted the baby on Plumpynut
- [ ] Preferred formula
- [ ] Other
- [ ] Prefer not to say

**61ma. Specify other reason your wife did not breastfeed your last baby**

______________________________

**62f. How soon after birth did you breastfeed the baby?**

- ○ Less than ½ hour
- ○ ½ hour to 1 hour
- ○ More than one hour but less than 24 hours
- ○ 24 hours-3 days
- ○ More than 3 days
- ○ Don't know/Don't remember
- ○ Other
- ○ Prefer not to say

**62fa. Specify other: how soon after birth for breastfeeding**

______________________________

**62m. How soon after birth did your wife breastfeed the baby?**

- ○ Less than ½ hour
- ○ ½ hour to 1 hour
- ○ More than one hour but less than 24 hours
- ○ 24 hours-3 days
- ○ More than 3 days
- ○ Don't know/Don't remember
- ○ Other
- ○ Prefer not to say

**62ma. Specify other: how soon after birth for breastfeeding**

______________________________

**63f. Why did you wait more than 3 days to breastfeed?**

- ☐ It is traditional to wait
- ☐ The early milk is not good for a baby
- ☐ My family advised against it
- ☐ I was too sick to breastfeed
- ☐ The TBA/CHW advised against it
- ☐ The clinic advised against it
- ☐ I had problems breastfeeding
- ☐ Don't know/Don't remember
- ☐ Other
- ☐ Prefer not to say

**63fa. Specify other why you waited more than 3 days to breastfeed**

______________________________

**63m. Why did your wife wait more than 3 days to breastfeed?**

- [ ] It is traditional to wait Other
- [ ] The early milk is not good for a baby
- [ ] My family advised against it
- [ ] I was too sick to breastfeed
- [ ] The TBA/CHW advised against it
- [ ] The clinic advised against it
- [ ] I had problems breastfeeding
- [ ] Don't know/Don't remember
- [ ] Other
- [ ] Prefer not to say

**63ma. Specify other why you waited more than 3 days to breastfeed**

______________________________

**64. During the first six months after the birth of your last child, did your child get food or water in addition to breastmilk?**

- ○ Yes
- ○ No
- ○ Don't know/Don't remember
- ○ Prefer not to say

**65. How long after the birth of your last child was the baby given something besides breastmilk?**

*(Enter a number only; the next question you will be able to enter hours, days, months or years; Enter "0" if nothing other than breastmilk in the first 6 months*

______________________________

**65a. Please select if the number just entered is days, months or years**

- ○ Hours
- ○ Days
- ○ Months
- ○ Years

**66. During the first 6 months after birth of your last baby, what else was given to the baby besides breastmilk?**
*(Check all that apply; Do not read)*

- [ ] Cow's milk
- [ ] Water
- [ ] Sorghum/rice porridge
- [ ] Goat's milk
- [ ] Formula
- [ ] Corn-soy porridge
- [ ] PlumpyNut
- [ ] Don't know/ Don't remember
- [ ] Other
- [ ] Prefer not to say

**66a. Specify other given to baby during the first 6 months besides breastmilk**

______________________________

**66b. Was your last baby given any type of powdered milk instead of or in addition to breastmilk such as Nido, Mamalac, Almudhish)**

- ( ) Yes
- ( ) No
- ( ) Don't know
- ( ) Prefer not to say

**67. Who in the community helps mothers' breastfeed if there is difficulty?**

- [ ] No one
- [ ] Mother/mother-in-law
- [ ] Relative
- [ ] Traditional birth attendant
- [ ] Nurse midwife
- [ ] Midwife
- [ ] Doctor
- [ ] Community health worker
- [ ] Don't know
- [ ] Other
- [ ] Prefer not to say

**67a. Specify other who helps with breastfeeding difficulties**

______________________________

**68f. Did you skip meals when you were breastfeeding your last child because it is polite to feed guests/your husband/your children first?**

- ◯ Yes
- ◯ No
- ◯ Don't know/Don't remember
- ◯ Prefer not to say

**68m. Did your wife skip meals when she was breastfeeding your last child because it is polite to feed guests/you/your children first?**

- ◯ Yes
- ◯ No
- ◯ Don't know/Don't remember
- ◯ Prefer not to say

**70. In your opinion, what are the signs your child with a respiratory infection needs to be seen immediately in a clinic or hospital?**

*(Check all listed; Do not read)*

- ☐ Could not list any
- ☐ Fast breathing
- ☐ Chest wall draws inward
- ☐ Fever
- ☐ Cough
- ☐ Difficulty breathing
- ☐ Don't know
- ☐ Other
- ☐ Prefer not to say

**70a. Specify other signs a child with a respiratory infection needs to be seen immediately**

______________________________

**71. I am pleased you could name some of these reasons, where did you learn about these?**

*(Check all listed)*

- [ ] My wife/ my husband
- [ ] Clinic
- [ ] TBA
- [ ] CHW
- [ ] Relative
- [ ] Mother/mother-in-law
- [ ] Radio
- [ ] Written material (newspaper, fliers, billboards)
- [ ] TV
- [ ] Mobile messages
- [ ] Don't know/Don't remember
- [ ] Other
- [ ] Prefer not to say

**71a. Specify other - where they learned about signs of child with respiratory infection**

_______________________________________________

**72. In your opinion, what are some of the signs that a child with Malaria should be seen immediately in the clinic or hospital?**

*(Check all listed; Do not read)*

- [ ] Could not list any
- [ ] Convulsions (fits)
- [ ] Unconsciousness
- [ ] Sleepiness
- [ ] Confusion
- [ ] Inability to walk, sit, speak or recognize relatives
- [ ] Repeated vomiting
- [ ] Can't eat or drink
- [ ] Small amount of urine/no urine/dark urine
- [ ] Severe diarrhea
- [ ] Bleeding from nose, gums, or other sites
- [ ] High fever
- [ ] Severe dehydration
- [ ] Pale skin (anemia)
- [ ] Yellow eyes
- [ ] Don't know
- [ ] Other
- [ ] Prefer not to say

**72a. Specify other signs a child with malaria should be seen**

______________________________

**73. I am pleased you could name some of these signs, where did you learn about these?**
*(Check all listed)*

- [ ] My wife/ my husband
- [ ] Clinic
- [ ] TBA
- [ ] CHW
- [ ] Relative
- [ ] Mother/mother-in-law
- [ ] Radio
- [ ] Written material (newspaper, fliers, billboards)
- [ ] TV
- [ ] Mobile messages
- [ ] Don't know/Don't remember
- [ ] Other
- [ ] Prefer not to say

**73a. Specify other: where they learned malaria signs**

______________________________________________

**74. In your opinion, what are some of the signs a child with diarrhea should be seen immediately in a clinic or hospital?**
*(Check all signs listed; Do not read)*

- [ ] Could not list any
- [ ] Skin is dry and doesn't look normal
- [ ] Does not want to eat or drink
- [ ] Mouth is dry
- [ ] Urine is decreased/dark
- [ ] Lethargic
- [ ] Sleepiness
- [ ] Convulsions/fits
- [ ] Eyes are sunken
- [ ] Yellow eyes
- [ ] Fast breathing
- [ ] Arms and legs are cold
- [ ] Don't know
- [ ] Other
- [ ] Prefer not to say

**74a. Specify other signs of child with diarrhea who should be seen immediately**

---

**75. I am pleased you could name some of these signs, where did you learn about these?**

*(Check all listed; Do not read)*

- [ ] My wife/ my husband
- [ ] Clinic
- [ ] TBA
- [ ] CHW
- [ ] Relative
- [ ] Mother/mother-in-law
- [ ] Radio
- [ ] Written material (newspaper, fliers, billboards)
- [ ] TV
- [ ] Mobile messages
- [ ] Don't know/Don't remember
- [ ] Other
- [ ] Prefer not to say

**75a. Specify other: learned diarrhea signs from?**

---

**76. In your opinion, what are some of the things you can do to avoid starvation or malnutrition in your children <5 years including newborns?**

*(Check all listed; Do not read)*

- [ ] Cannot list any
- [ ] Good pregnancy weight
- [ ] Eating well during pregnancy
- [ ] Taking appropriate vitamins
- [ ] Taking iron
- [ ] Getting enough micronutrients while pregnant from foods like leafy green vegetables
- [ ] Immunizations
- [ ] Deworming
- [ ] Seeking help if baby gets thin
- [ ] Breastfeeding
- [ ] Using bed nets
- [ ] Checking my baby weight
- [ ] Don't know/Don't remember
- [ ] Other
- [ ] Prefer not to say

**76a. Specify other things that can be done to avoid malnutrition or starvation in children less than 5 years old including newborns?**

________________________________________

**77. I am pleased you could name some of these things, where did you learn about these?**

*(Check all listed; Do not read)*

- [ ] My wife/ my husband
- [ ] Clinic
- [ ] TBA
- [ ] CHW
- [ ] Relative
- [ ] Mother/mother-in-law
- [ ] Radio
- [ ] Written material (newspaper, fliers, billboards)
- [ ] TV
- [ ] Mobile messages
- [ ] Don't know/Don't remember
- [ ] Other
- [ ] Prefer not to say

**77a. Specify other: learned signs from?**

______________________________

**78. Do you have children under 2 years of age in this household?**

- ( ) Yes
- ( ) No
- ( ) Prefer not to say

**78a. DATA COLLECTOR: ask to see immunization cards for all children under 2 years of age: Do ALL children (under 2 years old) have 3 doses of DPT or Pentavac?**

- ( ) Yes
- ( ) No
- ( ) No immunization cards available
- ( ) Did not want to present cards

DATA COLLECTOR: Be sure there is no one else listening to the interview and you are alone with the respondent - if there are others, politely as them to leave so you can continue the survey.

______________________________

**79. Who makes the decision about you going to the clinic or hospital?**

- I make this decision
- My spouse/partner
- Other relative
- Both my spouse/partner and myself (joint decision)
- Mother or Mother-in-law
- I don't go to the clinic or the hospital
- There is no clinic or hospital
- Other
- Don't know
- Prefer not to say

**79a. Specify other who makes decision for going to clinic or hospital**

______________________________

**80. Who makes the decision about your children going to the clinic or hospital?**

- I make this decision
- My spouse/partner
- Other relative
- Both my spouse/partner and myself (joint decision)
- Mother or Mother-in-law
- I don't go to the clinic or the hospital
- There is no clinic or hospital
- Other
- Don't know
- Prefer not to say

**80a. Specify other who makes decision for children going to a clinic or hospital**

______________________________

**81. Who makes financial decisions in your household?**

- I make this decision
- My spouse/partner
- Other relative
- Both my spouse/partner and myself (joint decision)
- Mother or Mother-in-law
- Other
- Don't know
- Prefer not to say

**81a. Specify other who makes financial decision in your household**

______________________________

**82.Who makes the decision about schooling for the children?**

- I make this decision
- My spouse/partner
- Other relative
- Both my spouse/partner and myself (joint decision)
- Mother or Mother-in-law
- Other
- Don't know
- Prefer not to say

**82a. Specify other who makes decision about schooling for children**

______________________________

DATA COLLECTOR: Be sure there is no one else listening to the interview and you are alone with the respondent - if there are others, politely as them to leave so you can continue the survey.

READ TO THE RESPONDENT: "Now I am going to read some statements to you, please tell me if you PERSONALLY agree or disagree with the statement I read. I need you to say "I agree'" or "I disagree", Not Yes or No.

| 83. Statements: | Agree | Disagree | Don't know | Prefer not to say |
|---|---|---|---|---|
| 83a. My spouse/partner has the right to have sex with me even if I don't want to | ○ | ○ | ○ | ○ |
| 83b. I have the right to refuse sex | ○ | ○ | ○ | ○ |

| | | | | |
|---|---|---|---|---|
| **83c. My spouse has the right to beat me if I disobey him/her or if I don't do things the right way** | ◯ | ◯ | ◯ | ◯ |
| **83d. My spouse would allow me to use birth control if Iwanted to use it** | ◯ | ◯ | ◯ | ◯ |
| **83e. My husband or I would use condoms if I asked him to** | ◯ | ◯ | ◯ | ◯ |
| **83f. My husband/I take(s) the children to the clinic for immunizations** | ◯ | ◯ | ◯ | ◯ |
| **83g. Consumption of safe and enough water can prevent waterborne diseases** | ◯ | ◯ | ◯ | ◯ |
| **83f. Defecating near water source can cause contamination** | ◯ | ◯ | ◯ | ◯ |
| **83i. Animal dung, if not properly managed, causes health problems** | ◯ | ◯ | ◯ | ◯ |
| **83j. Diarrheal diseases are caused by poor personal hygiene and sanitation** | ◯ | ◯ | ◯ | ◯ |
| **83k. Washing hand after using latrine prevents diarrheal diseases** | ◯ | ◯ | ◯ | ◯ |
| **83l. It is good to feed a child different types of food each day** | ◯ | ◯ | ◯ | ◯ |
| **83m. Early breastmilk protects newborn babies from infection** | ◯ | ◯ | ◯ | ◯ |
| **83n. Babies should be breastfed exclusively for the first 6 months** | ◯ | ◯ | ◯ | ◯ |
| **83o. Infants and children should be weighed on a regular basis at the clinic** | ◯ | ◯ | ◯ | ◯ |

**84. Where have you heard health and hygiene messages?**

- ☐ I have not heard any
- ☐ At the clinic from staff
- ☐ Radio
- ☐ TV
- ☐ Mobile phone
- ☐ Billboards- Written materials
- ☐ Visits from healthcare providers
- ☐ Community outreach volunteers
- ☐ Community members
- ☐ Relatives
- ☐ Don't know/Don't remember
- ☐ Other
- ☐ Prefer not to say

**84a. Specify other: where health messages are heard**

---

**85. What would be the best way to get health messages to you?**
*(Write what is said in the box; put "99" if they prefer not to say)*

---

**86. Do you own?**

| | Yes | No | Prefer not to say |
|---|---|---|---|
| **TV** | ○ | ○ | ○ |
| **Radio** | ○ | ○ | ○ |
| **Mobile Phone** | ○ | ○ | ○ |

**87. Have any of your children been screened for malnutrition (where the arm was measured) at home by a CHW, TBA or other healthcare provider?**

- Yes
- No
- Don't know/Don't remember
- Prefer not to say

**88. Have you received education on feeding such as breastfeeding or about when to add solid food for your infant/child(ren)?**

- Yes
- No
- Don't know/Don't remember
- Prefer not to say

**89. Have any of your children been treated for malnutrition that required a feeding tube or had to be treated at a clinic ?**

- Yes
- No
- Don't know/Don't remember
- Prefer not to say

**90. In your opinion, when should you wash your hands?**
*(Do not read; Check all listed)*

- [ ] After defecation/using the toilet
- [ ] Before eating
- [ ] After changing a diaper or cleaning a child's bottom
- [ ] Before preparing food
- [ ] Before feeding an infant
- [ ] Other
- [ ] Prefer not to say
- [ ] Don't know/Don't remember

**90a. Specify other: when you should wash your hands**

______________________________

**91. I am pleased you could name when you should wash your hands, where did you learn about this?**
*(Check all listed; Do not read)*

- [ ] My wife/ my husband
- [ ] Clinic
- [ ] TBA
- [ ] CHW
- [ ] Relative
- [ ] Mother/mother-in-law
- [ ] Radio
- [ ] Written material (newspaper, fliers, billboards)
- [ ] TV
- [ ] Mobile messages
- [ ] Don't know/Don't remember
- [ ] Other
- [ ] Prefer not to say

**91a. Specify other: when you should wash your hands**

______________________________

**92. Where do you store your water?**

*(Check all listed; DATA COLLECTOR - you may need to ask to see the container(s))*

- [ ] Sealed or covered bucket
- [ ] Uncovered bucket
- [ ] Jerry can with a top (cover)
- [ ] Jerry can without top (cover)
- [ ] Covered pot
- [ ] Uncovered pot
- [ ] Other
- [ ] Prefer not to say

**92a. Specify other container**

______________________________

**93. Data collector – ask to see water storage container and determine if the inside and outside are free of dirt or sediment, foreign objects.**

- ○ Yes
- ○ No
- ○ No water storage container
- ○ Prefer not to say

**94. Are you using tablets to clean your water that you use for drinking cooking and hygiene?**

- ○ Yes
- ○ No
- ○ Don't know/Don't remember
- ○ Prefer not to say

**95. Please show me your water purification tablets so I can write down what you are using.**

- [ ] NaDCC tablets
- [ ] Sodium hypochlorite (e.g. Aquatabs or Water Guard)
- [ ] Combined flocculant/disinfectant sachets
- [ ] Chlorine solution
- [ ] Other
- [ ] No tablets available to see (ran out of tablets)

**95a. Specify other tablets/solution being used to clean water**

______________________________

**96. Has where you collect your water been repaired and cleaned in the last 3 months?**

- Yes
- No
- Don't know/Don't remember
- Prefer not to say

**97. Are you a member of a water user committee?**

- Yes
- No
- Don't know/Don't remember
- Prefer not to say

**98. Does your community have a water user committee?**

- Yes
- No
- Don't know/Don't remember
- Prefer not to say

| 99. Does/did your water committee have the following: | Yes | No | Don't know/Unsure | Prefer not to say |
|---|---|---|---|---|
| Water safety plan | | | | |
| Training | | | | |
| Active committee | | | | |

| 100. In your household, do you have? | Yes | No | Don't know | Prefer not to say |
|---|---|---|---|---|
| Pit latrine | | | | |
| A handwashing station | | | | |
| Soap | | | | |

**101. What is your ethnicity?**

- ○ Dinka
- ○ Nuer
- ○ Murle
- ○ Shiluk
- ○ Anyuak
- ○ Other
- ○ Prefer not say

**101a. Specify other ethnic group**

______________________________

**[CLOSING STATEMENT FOR ALL PARTICIPANTS] Thank you for taking the time to talk with me. As I stated earlier, this information will be kept confidential, and we are gathering this information to learn how to better protect the health of South Sudanese IDPs, returnees and the host community. As we move around the State conducting this survey, we are telling people that this is a Health Survey and we are not telling anyone who we are interviewing. We know that people in this area will be curious about all of us and wondering what we are doing here. We suggest that you do not give details about what we discussed, and that you simply say that it is a survey about health. Again, thank you for your time. Do you have any questions or comments?**

hh:mm
______________________________
