# Supplementary material for: A mixed-methods nutrition, water, sanitation and hygiene knowledge, attitudes, and practices survey of IDPs, returnees, and host community members in four counties of Jonglei state, South Sudan
Source: J Health Popul Nutr. 2025 Feb 25;44:48. doi: 10.1186/s41043-025-00789-3 (PMC11863811; doi:10.1186/s41043-025-00789-3)
Supplement: Supplementary file 3 — Additional file 3. [file 41043_2025_789_MOESM3_ESM.pdf]

JESI-KII Partners NGOs Gov Traditional Authority (Supervisors Only)

1. Enter a date and time

yyyy-mm-dd hh:mm

2. Data Collector ID

- Francis
- Ann
- Lazarus
- David

2a. Interview ID

3. The sex of the respondent is?

- Male
- Female

4. County

- Pigi Canal
- Fangak
- Ayod
- Nyirol

4a. Pigi Canal Payam

- Canal

4b. Ayod Payam

- Mogok

4c. Nyirol Payam

- Pultruk
- Pading

4d. Fangak Payam

- Pagwir
- Toch

5. Mogok Boma

- Mogok-Panyang
- Gar
- Kurwai

5a. Pultruk Boma

- Bariak

5b. Pading Boma

- Guer

5c. Canal Boma

- Pigi
- Korfulus
- Mat

5d. Pagwir Boma

- Pagwir

5e. Toch Boma

- Tock

CONSENT STATEMENT

Read the entire statement to the person you are interviewing

We are working with IMA, a non-governmental organization from the United States. IMA has been working in South Sudan on many projects. IMA also works in other countries with the support of many governments, private donors and other international organizations to help them reach as many people as possible. However, they do not support any political or religious group.

We are conducting a survey to understand the health and needs of internally displaced persons, returnees and community members hosting the internally displaced persons in your area. We have asked permission from your community leaders to conduct this survey. We will read questions to you and ask for answers to each question. The findings of this study will be used to try to improve health in your community especially of pregnant women, babies and children. We are not here to provide humanitarian assistance at this time. Your participation is voluntary. Your answers may not directly benefit you, but they will help organizations and the government to plan services for you, your family, and your community. We realize that many people have suffered greatly and may have much to tell. But this survey requires only BRIEF responses to a limited number of questions. We expect this will take about 30 minutes of your time.

You do not need to give us your name. None of your answers will be shared with anyone else. It will not be possible to personally identify your answers. We do not feel that this survey puts you at risk, however they may be risk that we are unaware of. If we felt there was risk, we would not have approached you at all.

Your participation in this study is voluntary. You may decide not to participate, or you may leave the study at any time. Your decision will not result in any penalty or loss of benefits to which you are otherwise entitled. Your alternative is not to participate in the survey.

You do not need to give us your name at all. None of your answers will be shared with anyone else and it will not be possible to know your answers from anyone else's answers. However, the researchers and the IRB referenced below will see your answers. Even if you do provide your name, it will not be kept with the answers to the survey questions to maintain your confidentiality. If you do not understand a question, please ask me to explain it to you. Please note that some questions that we ask are sensitive, therefore it is important that we have some privacy for our conversation. You are free to stop at any time during the interview. If a question makes you uncomfortable, we will skip the question and go to the next question. If you have any questions, concerns, or complaints about the survey, or if you feel that participating in the research has caused you harm, you can get more information from your interviewer .

This research is being overseen by an Institutional Review Board ("IRB"). An IRB is a group of people who perform independent review of research studies. Your community health worker will be able to help you reach them if you have questions, concerns, or complaints that are not being answered by the research team, you are not getting answers from the research team, you cannot reach the research team, you want to talk to someone else about the research, or if you have questions about your rights as a research subject.

---

6. May I ask you some questions?

○ Yes ○ No

7. Why do you refuse to participate in the survey?

- Lacks time
- Fears reprisal
- Tired of doing surveys
- Nothing has changed with these surveys
- Doesn't feel well
- No response
- Other

7a. Other reason -refusal to participate

____

8. Thank you for your time, we are sorry you are unwilling or unable to participate. I will respect your desire not to participate, can you please tell me how old you are before I leave? (years).

Enter "99" if they refuse to say their age

____

END SURVEY

Data Collector: check "Go to end" and move to the next household

hh:mm

9. Do you know your age in years?

- Yes
- No
- Prefer not to say

9a. How old are you? (years)

(if they refuse to answer put "99")

____

10. Do you have a disability?

- Yes
- No
- Don't know
- Prefer not to say

10a. What type or types of disability do you have?

- [ ] Vision
- [ ] Hearing
- [ ] Mobility
- [ ] Cognition (Remembering)
- [ ] Self-Care
- [ ] Communication
- [ ] None of the above
- [ ] Don't know

11. In your opinion, what keeps people in your community from accessing healthcare?
Probe for beliefs, knowledge, traditional practices, conflict, poverty others...

---

12. In your opinion what keeps women from seeking care when they are pregnant?
Probe for beliefs, knowledge, home duties, traditional practices, conflict. GBV, poverty others...

---

13. In your opinion what keeps children from being seen at clinics for care?
Probe for beliefs, knowledge, home duties, traditional practices, conflict. GBV, poverty others...

---

14. What do you think causes malnutrition in this community?
Probe for beliefs, knowledge, home duties, traditional practices, conflict. GBV, poverty others...

---

15. How effective have NGOs been in the last year in addressing women's health?
Probe for barriers, gaps

---

16. How effective have NGOs been in the last year in addressing child health?
Probes for barriers gaps

---

17. How effective has the government been effective in getting care to women and children in your community?
Probe for barriers, gaps

---

18. Where is the best place to get health information to the community?

---

19. How have traditional practices influenced (bad or good) health care for women and children?

---

20. What are the greatest needs of the community?

---

21. Anything you want to say that I did not ask?

---

22. What is your ethnicity?

- ○ Dinka
- ○ Nuer
- ○ Murle
- ○ Shiluk
- ○ Anyuak
- ○ Other
- ○ Prefer not to say

22a. Specify other ethnic group

---

23. Data Collector: ADD specifics about this respondent:

- ☐ Organization (specify)
- ☐ Government (specify)
- ☐ Other Partner (specify)
- ☐ Healthcare Worker
- ☐ Community Health Worker
- ☐ South Sudan Red Cross
- ☐ Traditional Authority - Witch Doctor
- ☐ Traditional Authority - Spirit Master

23a. Specify Organization
Organization name (do not name the person)

23b. Specify Government
Government office (do not name person)

23c. Specify Other Partner

[CLOSING STATEMENT FOR ALL PARTICIPANTS] Thank you for taking the time to talk with me. As I stated earlier, this information will be kept confidential, and we are gathering this information to learn how to better protect the health of South Sudanese IDPs, returnees and the host community. As we move around the State conducting this survey, we are telling people that this is a Health Survey and we are not telling anyone who we are interviewing. We know that people in this area will be curious about all of us and wondering what we are doing here. We suggest that you do not give details about what we discussed, and that you simply say that it is a survey about health. Again, thank you for your time. Do you have any questions or comments?

hh:mm

____

24. Other comments

____
