# Supplementary material for: A mixed-methods nutrition, water, sanitation and hygiene knowledge, attitudes, and practices survey of IDPs, returnees, and host community members in four counties of Jonglei state, South Sudan
Source: J Health Popul Nutr. 2025 Feb 25;44:48. doi: 10.1186/s41043-025-00789-3 (PMC11863811; doi:10.1186/s41043-025-00789-3)

**Additional File IV**

A mixed-methods nutrition, water, sanitation and hygiene knowledge, attitudes, and practices survey of IDPs, returnees, and host communities in four counties of Jonglei State, South Sudan

## Table of Contents

**Table 1: Sample population estimates by Boma Among 859 Households in Jonglei South Sudan, June-July 2023** ........ 3
**Table 2: Demographic characteristics of the WASH and nutrition KAP study participants among 859 Households in Jonglei South Sudan, June-July 2023** ........ 4
**Table 3: Weighted Odd Ratios for Knowledge of Handwashing Reasons Among 859 Households in Jonglei South Sudan, June-July 2023** ........ 6
**Table 4: Characteristics of qualitative interviews quoted for the WASH and nutrition KAP study among 859 Households in Jonglei South Sudan, June-July 2023** ........ 7
**Table 5: Weighted Odds Ratios for Positive Nutrition Attitudes Among 859 Households in Jonglei South Sudan, June-July 2023** ........ 7
**Table 6:Weighted Odds Ratios for Exclusive Breastfeeding Among 859 Households in Jonglei South Sudan, June-July 2023** ........ 9

**Table 1: Sample population estimates by Boma** Among 859 Households in Jonglei South Sudan, June-July 2023

| County | Payam | Boma | Population Host and IDP/HH # | Population Proportion/# surveys[a] | IDP population | Estimated IDP population[b] | n[c] (% per Boma) | Weight | Weighted n |
|---|---|---|---|---|---|---|---|---|---|
| Ayod | Mogok | Mogok-Panyang | 27,113/3127 | 60%/135 | 15,949 | 15,464 | 135 (60%) | 1.15 | 156 |
| | | Gar | 2,445/282 | 5%/11 | 200 | 2,445 | 11 (5%) | 1.28 | 14 |
| | | Kurwai | 15,849/1828 | 35%/79 | 9,323 | 5,417 | 79 (35%) | 1.15 | 91 |
| | | Total Population | 45,407/5,237 | 100%/225 | 25,472 | 23,326 | 225 (100%) | -- | -- |
| Nyirol | Pultruk | Bariak | 22,440/2,588 | 59%/135 | 13,200 | 1,951 | 115 (61%) | 1.12 | 129 |
| | Pading | Guer | 15,342/1,770 | 41%/94 | 9,025 | 1,451 | 74 (39%) | 1.19 | 88 |
| | | Total Population | 37,782/4,358 | 100%/229 | 22,225 | 3,402 | 189 (100%) | -- | -- |
| Pigi | Canal | Canal | 9,333/1,076 | 22%/61 | 5,490 | 2,142 | 61 (28%) | 0.88 | 54 |
| | | Korfulus | 25,551/2,947 | 61%/123 | 15,030 | 7,686 | 123 (56%) | 1.19 | 147 |
| | | Mat | 7,155/825 | 17%/37 | 4,209 | 967 | 37 (17%) | 1.11 | 41 |
| | | Total Population | 42,039/4,849 | 100%/221 | 24,549 | 10,795 | 221 (100%) | -- | -- |
| Fangak | Pagwir | Chortbora | 13,600/1754 | 63%/63[d] | 1,608 | 8,830 | 62 (28%) | 1.41 | 87 |
| | Toch | Toch | 9,010/1,039 | 37%/162 | 5,300 | 5,061 | 162 (72%) | 0.32 | 52 |
| | | Total Population | 22,610/2,608 | 100%/225 | 6,908 | 13,891 | 224 (100%) | -- | -- |
| | | Total 4 Counties | 149,446/17,237 | 100%/900 | 79,334 | 51,414[e] | 859 | | 859 |

[a] Number of surveys conducted includes non-respondents
[b] Estimated IDP population based on the sample proportion of respondents identifying as IDP within each boma
[c] Number of complete surveys/consenting respondents
[d] Proportional sampling was not implemented in Fangak due to flooding
[e]The total estimated IDP is based on the calculating the estimated number of IDPs in each boma and then summing it up. In the box below, it is calculated by taking the estimated population (149,446) divided by the average number of household members (8.7) and then taking the sample proportion of 37% IDP households to get 6,378 households

A total of 859 households consented to the study which was composed of IDPs (37%), returnees (18%), and host community members (45%) with response rates of 98% among IDPs and returnees and 90% for host community households. The systematic random selection of households allowed for estimation of the population of IDPs, returnees, and host community members in the project area. Across the 10 bomas, there are an estimated 6,378 IDP, 3,103 returnees, and 7,757 host community households.

**Table 2: Demographic characteristics of the WASH and nutrition KAP study participants among 859 Households in Jonglei South Sudan, June-July 2023**

| | N | All | IDPs | Returnees | Host Community | p-Value[a] | p-Value[a] |
|---|---|---|---|---|---|---|---|
| | | Weighted % (95% CI) | Weighted % (95% CI) | Weighted % (95% CI) | Weighted % (95% CI) | IDP vs Host Community | Returnee vs Host Community |
| Sex | | | | | | 0.254 | 0.483 |
| Female | 586 | 68·1 (64·7-71·3) | 70·2 (64·4-75·4) | 69·2 (61·2-76·3) | 66·1 (61·0-70·9) | | |
| Male | 273 | 31·9 (28·7-35·3) | 29·8 (24·6-35·6) | 30·8 (23·7-38·8) | 33·9 (29·1-39·0) | | |
| Response rate | | | | | | | |
| Female | 610 | 95·9 (94·0-97·4) | 98·2 (95·3-99·5) | 99·1 (94·9-100·0) | 92·8 (88·9-95·6) | 0·011 | 0·031 |
| Male | 290 | 93·8 (90·2-96·3) | 99·0 (94·4-100·0) | 97·9 (88·7-99·9) | 88·3 (81·4-93·3) | 0·004 | 0·098 |
| Household Composition | | | | | | | |
| Total population (No·, range) | 849 | 8·7 (8·3-9·0) | 8·3 (7·9-8·8) | 8·3 (7·5-9·1) | 9·1 (8·6-9·6) | 0·022 | 0·085 |
| Children <5 years | 768 | 2·3 (2·2-2·4) | 2·4 (2·2-2·5) | 2·3 (2·1-2·4) | 2·2 (2·1-2·3) | 0·163 | 0·700 |
| Children <2 years | 738 | 1·2 (1·1-1·2) | 1·2 (1·1-1·3) | 1·1 (1·0-1·2) | 1·2 (1·1-1·2) | 0·267 | 0·665 |
| Ethnic Group | | | | | | <0·001 | 0·118 |
| Nuer | 640 | 72·7 (69·4-75·7) | 79·9 (74·5-84·3) | 68·8 (60·7-75·8) | 68·9 (63·9-73·5) | | |
| Dinka | 193 | 24·8 (21·9-28·0) | 17·7 (13·5-22·8) | 30·4 (23·4-38·4) | 27·8 (23·4-32·7) | | |
| Murle | 5 | 0·4 (0·1-1·2) | 0·7 (0·2-2·3) | 0·9 (0·1-6·0) | 0·0 (0·0-0·0) | | |
| Shiluk | 6 | 0·4 (0·2-1·1) | 0·1 (0·0-0·8) | 0·0 (0·0-0·0) | 0·8 (0·3-2·3) | | |
| Anyuak | 3 | 0·4 (0·1-1·3) | 1·0 (0·2-3·8) | 0·0 (0·0-0·0) | 0·1 (0·0-0·6) | | |
| Preferred not to say | 12 | 1·4 (0·7-2·5) | 0·7 (0·2-2·8) | 0·0 (0·0-0·0) | 2·4 (1·2-4·6) | | |
| Knows age (years) | | | | | | | |
| Female | 586 | 72·4 (68·3-76·1) | 77·1 (70·4-82·6) | 75·5 (65·9-83·0) | 67·3 (61·0-73·0) | <0·001 | 0·036 |
| Male | 273 | 77·4 (71·7-82·3) | 84·0 (74·1-90·5) | 77·5 (62·6-87·7) | 73·2 (64·4-80·4) | 0·103 | 0·963 |
| Age (years) | | | | | | | |
| Female | 483 | 31·0 (29·9-32·1) | 33·0 (31·1-35·0) | 30·0 (28·1-31·9) | 29·7 (27·9-31·4) | 0·016 | 0·958 |
| Male | 235 | 36·0 (34·6-37·4) | 35·6 (33·3-37·9) | 34·9 (32·5-37·3) | 36·8 (34·6-39·0) | 0·820 | 0·314 |
| Formal Education (years) | | | | | | | |
| Female | 569 | 0·6 (0·4-0·8) | 0·5 (0·3-0·8) | 0·4 (-0·1-1·0) | 0·8 (0·5-1·0) | 0·229 | 0·239 |
| Male | 268 | 2·3 (1·6-2·9) | 2·4 (1·0-3·7) | 2·3 (1·2-3·5) | 2·2 (1·2-3·2) | 0·957 | 0·899 |
| Self-reported literacy | | | | | | | |
| Female | 586 | 12·0 (9·5-15·0) | 9·7 (6·2-14·9) | 9·9 (5·4-17·4) | 14·6 (10·7-19·6) | 0·249 | 0·574 |
| Male | 273 | 27·2 (22·0-33·1) | 24·3 (16·1-34·8) | 36·9 (23·9-52·2) | 25·6 (18·6-34·2) | 0·813 | 0·119 |
| Verified literacy | | | | | | | |
| Female | 77 | 7·5 (5·6-10·1) | 7·7 (4·6-12·7) | 6·9 (3·4-13·7) | 7·7 (5·0-11·7) | 0·126 | 0·281 |
| Male | 77 | 16·9 (12·7-22·0) | 15·6 (9·2-25·3) | 19·1 (10·0-33·5) | 16·8 (11·2-24·6) | 0·333 | 0·677 |
| Verified literacy by years of education | | | | | | | |
| < 4 years | | | | | | | |
| Female | 32 | 2·7 (1·5-4·6) | 1·7 (0·5-5·3) | 6·7 (3·1-13·9) | 1·7 (0·6-4·8) | 0·328 | 0·021 |
| Male | 23 | 4·9 (2·6-9·0) | 3·6 (0·9-13·6) | 8·6 (2·6-24·6) | 4·3 (1·7-10·7) | 0·535 | 0·994 |
| ≥ 4 years | | | | | | | |
| Female | 44 | 67·8 (52·1-80·3) | 80·8 (53·7-93·9) | 48·4[e] | 61·7 (41·2-78·8) | 0·178 | 0·495 |
| Male | 54 | 61·9 (47·7-74·3) | 58·2 (33·9-79·1) | 52·2 (22·0-80·8) | 69·3 (46·8-85·3) | 0·334 | 0·366 |
| Self-reported numeracy | | | | | | | |
| Female | 586 | 20·7 (17·4-24·4) | 13·3 (9·1-19·0) | 33·0 (24·5-42·8) | 21·2 (16·5-26·9) | 0·078 | 0·002 |
| Male | 273 | 35·5 (29·8-41·7) | 36·9 (27·1-48·0) | 37·4 (24·3-52·7) | 33·9 (25·9-42·9) | 0·910 | 0·654 |
| Verified numeracy | | | | | | | |
| Female | 125 | 13·6 (10·9-16·8) | 9·4 (5·9-14·7) | 19·0 (12·5-27·8) | 14·5 (10·6-19·6) | 0·654 | 0·124 |
| Male | 98 | 27·1 (21·9-33·0) | 25·3 (16·9-35·9) | 37·4 (24·3-52·7) | 24·5 (17·6-33·0) | 0·464 | 0·039 |
| Verified numeracy by years of education | | | | | | | |
| < 4 years | | | | | | | |
| Female | 68 | 8·9 (6·7-11·9) | 4·2 (2·0-8·5) | 16·7 (10·4-25·8) | 9·4 (6·0-14·3) | 0·627 | 0·588 |
| Male | 41 | 15·0 (10·5-20·9) | 15·4 (8·2-27·0) | 20·7 (9·9-38·5) | 12·8 (7·3-21·3) | 0·276 | 0·077 |
| ≥ 4 years | | | | | | | |
| Female | 46 | 60·2 (44·4-74·1) | 65·7 (38·1-85·7) | 48·4[e] | 57·9 (37·5-75·9) | 0·764 | 0·735 |
| Male | 56 | 73·6 (59·7-84·0) | 62·2 (37·4-81·9) | 90·6 (48·3-99·0) | 74·1 (51·6-88·5) | 0·150 | 0·141 |
| Contributes to household income | | | | | | | |
| Female | 580 | 74·4 (70·5-78·0) | 61·5 (54·3-68·3) | 73·7 (64·1-81·5) | 85·0 (80·0-88·9) | <0·001 | 0·006 |
| Male | 271 | 85·7 (80·7-89·5) | 78·3 (68·3-85·9) | 88·3 (74·1-95·2) | 89·5 (82·5-93·9) | 0·015 | 0·793 |
| Weekly household income (USD)[b] | | | | | | | |
| Female | 159 | 70 | Median = 80 | 45 | 70 | 0·227 | 0·683 |
| Male | 118 | 50 | Median = 50 | 20 | 80 | 0·264 | 0·003 |
| Household owns land | 854 | 76·6 (73·4-79·4) | 72·0 (66·3-77·1) | 74·4 (66·5-81·0) | 80·7 (76·3-84·5) | 0·007 | 0·103 |

| | | | | | | | |
|---|---|---|---|---|---|---|---|
| Occupation | 854 | | | | | <0·001 | 0·302 |
| Agriculture/Herding/Fishing | | 53·9 (50·3-57·4) | 43·7 (37·8-49·8) | 53·8 (45·6-61·8) | 61·5 (56·3-66·4) | | |
| Other[c] | | 24·7 (21·7-27·9) | 23·6 (18·8-29·2) | 27·7 (21·0-35·7) | 24·2 (20·0-28·9) | | |
| Not working | | 12·9 (10·8-15·4) | 22·9 (18·3-28·3) | 7·9 (4·6-13·4) | 7·6 (5·3-10·7) | | |
| Small business | | 8·5 (6·7-10·7) | 9·8 (6·7-14·0) | 10·5 (6·4-16·7) | 6·8 (4·6-9·9) | | |
| Marital Status | 856 | | | | | 0·006 | 0·649 |
| Never married | | 3·3 (2·2-4·8) | 2·0 (0·9-4·5) | 3·9 (1·8-8·3) | 4·0 (2·4-6·7) | | |
| Married | | 86·3 (83·6-88·5) | 91·1 (86·9-94·0) | 84·6 (77·9-89·6) | 83·4 (79·1-86·9) | | |
| With a partner | | 4·4 (3·2-6·2) | 1·3 (0·4-3·6) | 6·3 (3·3-11·7) | 6·1 (4·0-9·1) | | |
| Wife/husband missing/conflict | | 1·7 (1·0-3·0) | 1·2 (0·4-3·6) | 0·7 (0·1-5·1) | 2·6 (1·3-4·9) | | |
| Divorced/separated/no longer in partnership | | 0·9 (0·4-1·8) | 1·6 (0·6-4·2) | 0·0 (0·0-0·0) | 0·7 (0·2-2·3) | | |
| Widow/widower | | 3·4 (2·3-5·0) | 2·9 (1·5-5·9) | 4·5 (2·1-9·2) | 3·3 (1·9-5·9) | | |
| Average number of wives (male) | 269 | 2·0 (1·7-2·2) | 1·8 (1·6-2·0) | 1·9 (1·5-2·3) | 2·1 (1·7-2·5) | 0·401 | 0·585 |
| Wife number (female)[d] | 539 | | | | | 0·007 | 0·065 |
| 1st | | 54·0 (49·7-58·3) | 57·2 (49·9-64·1) | 50·4 (40·6-60·2) | 53·2 (46·8-59·4) | | |
| 2nd | | 24·2 (20·8-28·1) | 22·4 (16·9-29·0) | 25·0 (17·4-34·5) | 25·4 (20·3-31·3) | | |
| 3rd or more | | 13·6 (10·9-16·8) | 17·5 (12·7-23·7) | 15·9 (9·8-24·7) | 9·6 (6·5-14·0) | | |
| Age at marriage | | | | | | | |
| Female | 446 | 17·9 (17·7-18·1) | 18·0 (17·6-18·4) | 18·2 (17·6-18·7) | 17·7 (17·4-17·9) | 0·791 | 0·654 |
| Male | 204 | 22·7 (22·0-23·4) | 21·0 (20·0-22·1) | 23·2 (22·0-24·5) | 23·7 (22·6-24·9) | <0·001 | 0·713 |
| Self-Reported Disability | | | | | | | |
| Female | 584 | 14·7 (11·9-18·0) | 9·8 (6·3-14·9) | 15·7 (9·8-24·3) | 18·2 (13·8-23·6) | 0·008 | 0·605 |
| Male | 273 | 21·7 (16·9-27·3) | 17·0 (10·1-27·3) | 20·1 (10·7-34·5) | 25·2 (18·2-33·9) | 0·079 | 0·774 |
| Type of Disability | 857 | | | | | | |
| Vision | | 6·1 (4·6-8·0) | 3·9 (2·1-7·1) | 8·2 (4·7-13·9) | 6·8 (4·6-10·0) | 0·095 | 0·587 |
| Hearing | | 3·0 (2·0-4·5) | 2·0 (0·9-4·7) | 4·1 (1·8-8·8) | 3·4 (1·9-5·8) | 0·285 | 0·698 |
| Mobility | | 4·8 (3·5-6·5) | 2·7 (1·3-5·5) | 3·3 (1·4-7·8) | 6·9 (4·6-10·0) | 0·013 | 0·113 |
| Cognition | | 0·7 (0·3-1·5) | 0·3 (0·0-2·1) | 0·0 | 1·2 (0·5-3·0) | 0·182 | 0·166 |
| Self-care | | 0·9 (0·4-1·8) | 1·3 (0·4-3·6) | 0·7 (0·2-3·4) | 0·6 (0·1-2·4) | 0·338 | 0·849 |
| Communication | | 1·3 (0·7-2·4) | 1·2 (0·4-3·7) | 1·6 (0·5-5·5) | 1·2 (0·5-3·2) | 0·997 | 0·704 |
| How long lived in the survey area (years) | 789 | 4·1 (3·8-4·5) | 3·3 (2·7-3·9) | 1·9 (1·5-2·4) | 5·9 (5·3-6·4) | <0·001 | <0·001 |
| Number of displacements over 2 years | | | | | | | |
| Female | 581 | 1·3 (1·2-1·4) | 1·5 (1·4-1·7) | 1·8 (1·6-2·0) | 0·9 (0·8-1·1) | <0·001 | <0·001 |
| Male | 272 | 1·4 (1·3-1·6) | 1·9 (1·6-2·1) | 1·8 (1·5-2·2) | 1·0 (0·8-1·2) | <0·001 | <0·001 |
| Top three ways for health messaging | 859 | | | | | | |
| Clinic | | 26·8 (23·8-30·1) | 20·8 (16·3-26·1) | 29·3 (22·5-37·2) | 30·3 (25·7-35·3) | 0·004 | 0·821 |
| CHW | | 29·3 (26·2-32·6) | 35·2 (29·6-41·2) | 30·2 (23·2-38·3) | 24·6 (20·4-29·4) | 0·002 | 0·178 |
| Media (Radio, TV, Phone) | | 12·2 (10·0-14·7) | 7·9 (5·2-11·9) | 14·8 (9·8-21·8) | 14·2 (10·9-18·3) | 0·009 | 0·851 |
| Owns | | | | | | | |
| TV | 838 | 8·1 (6·3-10·3) | 10·9 (7·6-15·4) | 9·0 (5·3-15·0) | 5·6 (3·6-8·6) | 0·011 | 0·154 |
| Radio | 840 | 13·7 (11·4-16·4) | 14·8 (10·9-19·6) | 17·0 (11·7-24·1) | 11·5 (8·6-15·3) | 0·214 | 0·088 |
| Mobile Phone | 838 | 40·0 (36·5-43·5) | 34·9 (29·3-40·9) | 53·9 (45·6-62·0) | 38·0 (33·0-43·3) | 0·394 | <0·001 |

Abbreviations: CI, confidence interval; USD, United States Dollar (1000 South Sudanese Pound = 1 USD)

[a] Categorical variables: Pearson $x^2$ test; Continuous variables: two tailed t-test for means.

[b] Unweighted medians for those who knew household income (n=277)

[c] Other occupation includes: Day labor, Government work, Homemaker, Fetching firewood, Hygiene prompter, Professional, Student, Cutting trees for sale, Hygiene prompter, NGO worker, Teacher

[d] Percentages calculated out of all women (including unmarried women) so column percentages do not add up to 100%

[e] Only two female returnees with at least 4 years of formal education

**Table 3: Weighted Odd Ratios for Knowledge of Handwashing Reasons Among 859 Households in Jonglei South Sudan, June-July 2023**

| | Weighted OR | 95% CI | p-value |
|---|---|---|---|
| IDP vs. Host | 1.00 | 0.97-1.04 | 0.887 |
| Returnee vs. Host | 0.98 | 0.93-1.03 | 0.409 |
| Male vs. Female | 0.99 | 0.95-1.04 | 0.758 |
| Younger age (16-25 yo) | 1.03 | 1.01-1.06 | 0.007 |
| Underage marriage (<18 yo) | 1.01 | 0.96-1.05 | 0.748 |
| Less than 4 years of formal education | 0.95 | 0.92-0.98 | 0.002 |
| Soap in the household | 0.99 | 0.97-1.01 | 0.238 |
| Pit latrine in the household | 0.95 | 0.92-0.98 | 0.004 |
| Handwashing station in the household | 0.89 | 0.84-0.95 | 0.001 |
| Boma | | | |
| Bariak (Reference) | 1.02 | 0.98-1.05 | 0.367 |
| Gar | 0.96 | 0.88-1.04 | 0.315 |
| Guer | 1.02 | 1.00-1.04 | 0.049 |
| Korfulus | 1.01 | 1.00-1.03 | 0.072 |
| Kurwai | 1.02 | 1.00-1.04 | 0.072 |
| Magok-Panyang | 1.03 | 1.00-1.07 | 0.048 |
| Mat | 0.89 | 0.82-0.97 | 0.006 |
| Chortbora | 1.01 | 0.99-1.03 | 0.170 |
| Pigi | 1.03 | 0.96-1.09 | 0.424 |
| Tock | 1.00 | (0.90-1.12) | 0.957 |

Adjusted odds ratios were estimated using a GLM with survey weights

**Table 4: Weighted Odds Ratios for Positive Nutrition Attitudes Among 859 Households in Jonglei South Sudan, June-July 2023**

| | Weighted OR | 95% CI | p-value |
|---|---|---|---|
| IDP vs. Host | 1.05 | 0.99-1.12 | 0.111 |
| Returnee vs. Host | 1.05 | 0.98-1.13 | 0.190 |
| Male vs. Female | 1.01 | 0.93-1.09 | 0.887 |
| Younger age (16-25 yo) | 0.96 | 0.90-1.02 | 0.206 |
| Underage marriage (<18 yo) | 1.00 | 0.94-1.07 | 0.950 |
| Less than 4 years of formal education | 0.93 | 0.87-0.99 | 0.030 |
| Second, third, or more wife | 0.99 | 0.93-1.05 | 0.788 |
| Children screened for malnutrition | 1.06 | 0.98-1.15 | 0.117 |
| Children treated for malnutrition | 0.93 | 0.88-0.98 | 0.008 |
| Boma | | | |
| Bariak (Reference) | | | |
| Guer | 1.05 | 0.99-1.11 | 0.110 |
| Korfulus | 1.01 | 0.95-1.07 | 0.793 |
| Kurwai | 0.63 | 0.55-0.72 | 0.000 |
| Magok-Panyang | 0.44 | 0.40-0.48 | 0.000 |
| Mat | 1.08 | 1.03-1.14 | 0.003 |
| Chortbora | 1.03 | 0.98-1.08 | 0.276 |
| Pigi | 1.04 | 1.00-1.08 | 0.056 |
| Tock | 1.03 | 0.98-1.07 | 0.236 |

Adjusted odds ratios for positive nutrition attitudes were estimated using a GLM with survey weights

**Table 5: Characteristics of qualitative interviews quoted for the WASH and nutrition KAP study among 859 Households in Jonglei South Sudan, June-July 2023**

| Key Informant Code | Sex | Boma | Title |
|---|---|---|---|
| DM1 | Female | Juba, Ayod | Survey manager, Nurse Midwife |
| DM2 | Msle | Juba, Nyirol | Survey manager |
| DM3 | Male | Juba, Pigi | Survey manager |
| DM4 | Male | Juba, Fangak | Survey manager |
| HC1 | Male | Ayod | Host community member |
| CM1 | Female | Ayod | Community member |

**Table 6: Weighted Odds Ratios for Exclusive Breastfeeding Among 859 Households in Jonglei South Sudan, June-July 2023**

| | Weighted OR | 95% CI | p-value |
|---|---|---|---|
| IDP vs. Host | 1.11 | 1.01-1.23 | 0.038 |
| Returnee vs. Host | 0.96 | 0.85-1.08 | 0.464 |
| Younger age (16-25 yo) | 0.99 | 0.90-1.09 | 0.869 |
| Underage marriage (<18 yo) | 0.99 | 0.91-1.09 | 0.908 |
| Less than 4 years of formal education | 1.05 | 0.95-1.17 | 0.340 |
| Educated on breastfeeding… | 1.18 | 1.03-1.35 | 0.015 |
| Second, third, or more wife | 0.96 | 0.88-1.04 | 0.300 |
| Children screened for malnutrition | 0.90 | 0.79-1.03 | 0.140 |
| Children treated for malnutrition | 0.91 | 0.78-1.05 | 0.197 |
| Able to identify at least one strategy to prevent starvation or malnutrition | 1.00 | 0.82-1.22 | 0.981 |
| Positive nutrition attitudes | 1.32 | 1.09-1.59 | 0.005 |
| Boma | | | |
| Bariak (Reference) | 1.00 | | |
| Gar | 1.29 | 0.83-2.00 | 0.251 |
| Guer | 0.71 | 0.55-0.92 | 0.011 |
| Korfulus | 1.37 | 1.15-1.65 | 0.001 |
| Kurwai | 1.01 | 0.80-1.27 | 0.918 |
| Magok-Panyang | 1.46 | 1.12-1.92 | 0.006 |
| Mat | 1.41 | 1.13-1.77 | 0.003 |
| Chortbora | 0.72 | 0.56-0.94 | 0.017 |
| Pigi | 1.40 | 1.16-1.70 | 0.001 |
| Tock | 0.78 | 0.64-0.95 | 0.015 |

Adjusted odds ratios for exclusive breastfeeding were estimated using a GLM with survey weights.

**Figure S1: Water Source in Ayod**

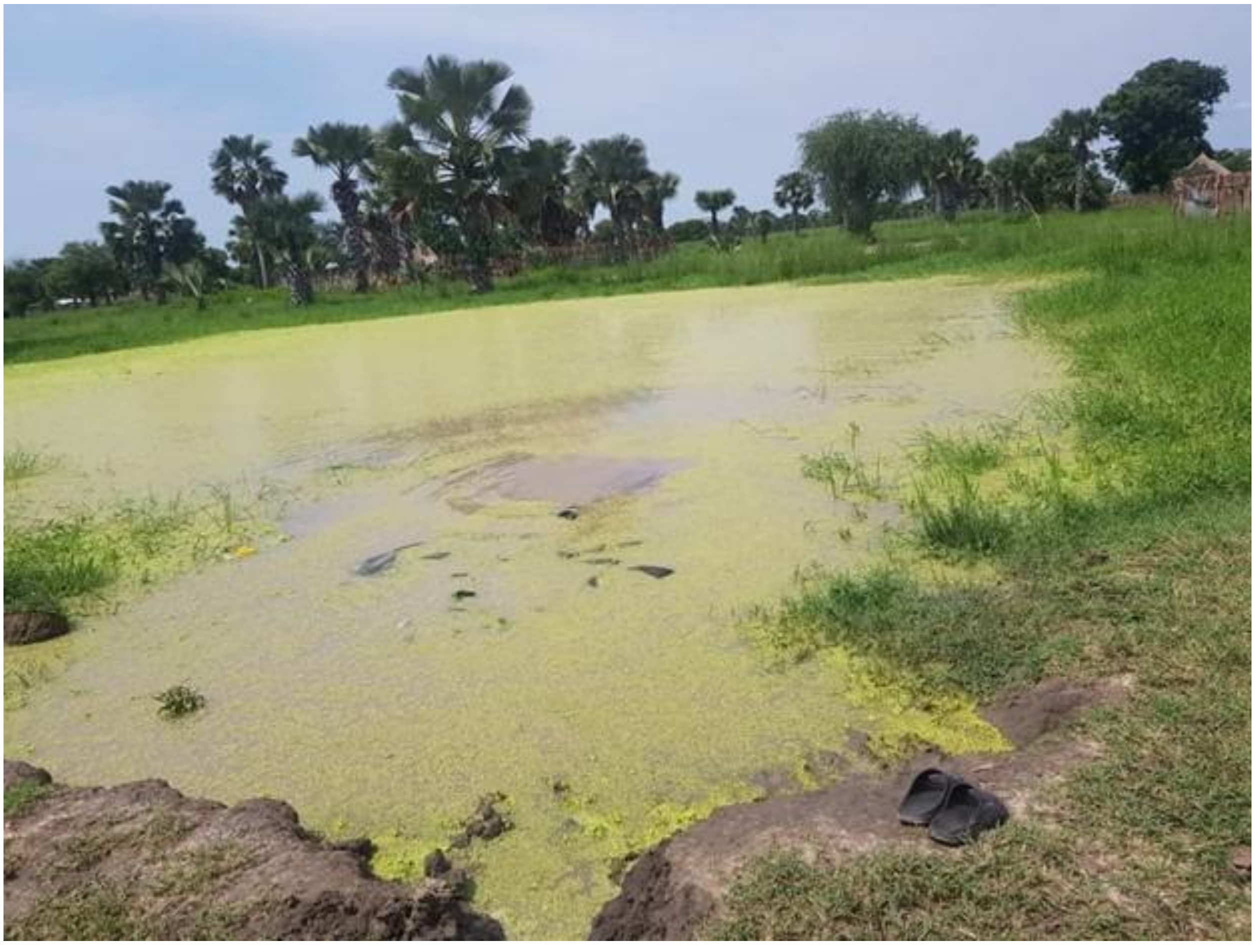

Supplement: Supplementary file 4 — Additional file 4. [file 41043_2025_789_MOESM4_ESM.pdf]
